# Biodegradable Zwitterionic PLA-Based Nanoparticles: Design and Evaluation for pH-Responsive Tumor-Targeted Drug Delivery

**DOI:** 10.3390/polym17182495

**Published:** 2025-09-16

**Authors:** Evi Christodoulou, Alexandros Tsimpolis, Konstantinos Theodorakis, Styliani Axypolitou, Ioannis Tsamesidis, Eleana Kontonasaki, Eleni Pavlidou, Dimitrios N. Bikiaris

**Affiliations:** 1 Department of Chemistry, Laboratory of Polymer Chemistry and Technology, Aristotle University of Thessaloniki, 54124 Thessaloniki, Greece; 2 Department of Basic Science, Medical School, University of Crete, 70013 Heraklion, Greece; altsimpolis@hotmail.com (A.T.); kostas_theodorakis@imbb.forth.gr (K.T.); 3 Institute of Molecular Biology and Biotechnology, Foundation for Research and Technology Hellas (FORTH), 70013 Heraklion, Greece; 4 Department of Prosthodontics, Faculty of Dentistry, School of Health Sciences, Aristotle University of Thessaloniki, 54124 Thessaloniki, Greece; stella.ax2@gmail.com (S.A.); johntsame@gmail.com (I.T.); kont@dent.auth.gr (E.K.); 5 Laboratory of Advanced Materials and Devices, Department of Physics, Faculty of Sciences, Aristotle University of Thessaloniki, 54124 Thessaloniki, Greece; elpavlid@auth.gr

**Keywords:** polylactic acid, poly(ethylene adipate), biodegradable polyesters, zwitterionic polymeric nanoparticles, pH-responsive drug delivery, paclitaxel

## Abstract

**Background/Objectives:** Biodegradable and pH-responsive nanocarriers using zwitterionic moieties represent a promising avenue for targeted delivery of chemotherapeutics. The present study addresses this by developing zwitterionic nanoparticles based on polylactic acid/poly(ethylene adipate) (PLA/PEAd) copolymers grafted with SBMA, designed to combine acid-triggered drug release with stealth-like biocompatibility. **Methods**: A series of polylactic acid/poly(ethylene adipate) (PLA/PEAd) copolymers with varying compositions (95/5, 90/10, and 75/25 *w*/*w*) were synthesized via ring-opening polymerization, followed by controlled radical grafting of the zwitterionic monomer [2-(Methacryloyloxy)ethyl]dimethyl-(3-sulfopropyl)ammonium hydroxide (SBMA), which was then successfully grafted upon their backbone. The resulting zwittenionic copolymers were thoroughly characterized for their structural and physicochemical properties, displaying tunable molecular weights of 3200–4900 g/mol, enhanced hydrophilicity and controlled degradation, with mass loss ranging from 8% to 83% over 30 days, depending on PEAd content and pH. Paclitaxel-loaded nanoparticles of spherical shape with sizes ranging from 220 to 565 nm were then fabricated. Drug release was pH-dependent with significantly higher release at pH 5.0 (up to ~79% for PLAPEAd7525-SBMA) compared to pH 7.4 (~18–35%). Hemolysis assays demonstrated excellent hemocompatibility, and cytotoxicity studies showed strong anticancer activity (>80% cell death in MDA-MB-231) with lower toxicity toward iMEFs, especially for PEAd-rich formulations. **Conclusions:** Our findings underline the potential of SBMA-functionalized PLA/PEAd nanoparticles as effective nano-carriers for tumor-targeted chemotherapy.

## 1. Introduction

Over the last decades, biodegradable polymeric nanocarriers have emerged as a powerful strategy in cancer therapy [1,2], offering a versatile platform with tunable properties that enables the smart targeted delivery of anticancer agents, thereby having tremendous potential to improve the therapeutic efficacy and safety of conventional chemotherapeutics with reduced off-target effects [3]. These nanoscale-engineered drug delivery systems (DDS) have several advantages, including the enhancement of drug solubility and bioavailability, the protection and stability of the encapsulated active ingredient against degradation, prolonged circulation times, and the potential for passive or active targeting of tumor tissues [4]. 

Biodegradable natural or synthetic polymers are enzymatically and/or non-enzymatically degraded when injected into the human body, leading to biocompatible and toxicologically safe by-products that are later eliminated by common metabolic pathways without secondary influence [5,6,7]. Examples of synthetic polymers commonly employed include poly(ester amides) [8], poly(amino acids) [9], poly(hydroxyalkanoates) [10,11], poly(orthoesters) [12], and polyesters. Especially the hydrolytically labile aliphatic polyesters prepared from either lactic or glycolic acid [13,14], or combinations of them (poly(lactide-co-glycolide), PLGA) [15,16], represent an attractive option that has been widely explored in such applications, with poly(lactic acid) (PLA) standing out for its biodegradability, processability, and excellent biocompatibility leading to FDA approval for safe use in biomedical applications [17]. However, PLA’s inherent hydrophobicity, its brittleness and slow biodegradation rate, often limit its utilization in areas requiring accelerated degradation rates, a case that is typically addressed by modification techniques or by copolymerizing/blending PLA with other polymers to improve its applicability [18]. One promising candidate for such modulation is poly(ethylene adipate) (PEAd), an aliphatic polyester synthesized from ethylene glycol and adipic acid and is often used as a plasticizer [19,20]. PEAd is biodegradable, renewable, and cost-effective, and has lower molecular weight values that can contribute to higher biodegradation rates. 

Furthermore, to achieve drug targeting and a desired pharmacological response at the tumor site, various available approaches based on polymeric DDSs have been reported [21,22,23]. Among them, pH-responsive polymeric nanoparticles have raised significant interest due to their ability to exploit the acidic microenvironment of tumors, which is typically characterized by a lower extracellular pH (~6.5–6.8) compared to normal tissues (~7.4), and even lower pH values (~5.0) within intracellular compartments such as endosomes and lysosomes [24,25,26]. This pH gradient can be utilized to trigger the release of encapsulated drugs selectively at the tumor site, thereby minimizing systemic toxicity and enhancing therapeutic effect. Such stimuli-responsive systems often incorporate pH-sensitive linkages or materials that undergo structural changes under mildly acidic conditions, i.e., swelling, degradation or charge conversion [27,28]. 

Within this context, zwitterionic polymers have very recently attracted great interest and further expanded the design possibilities of pH-responsive DDSs [29,30]. Zwitterionic materials, characterized by the presence of both positive and negative charges within the same repeat unit, exhibit remarkable antifouling properties, minimizing nonspecific interactions with biological components such as proteins and cells [31]. This translates into prolonged systemic circulation, improved pharmacokinetics, and enhanced biocompatibility [32], therefore such polymers can serve as superior alternatives to traditional hydrophilic coatings like polyethylene glycol (PEG) [33,34]. [2-(methacryloyloxy)ethyl]dimethyl-(3-sulfopropyl)ammonium hydroxide (SBMA) stands out due to its exceptional hydrophilicity and its ability to form dense, stable zwitterionic brushes when grafted onto hydrophobic polymer backbones [35]. Our group has previously published several works that underline the unique properties arising from integrating SBMA into polymeric drug delivery systems, which demonstrated pH-responsiveness, providing an additional layer of control over drug release [36,37]. 

In the present study, we present a series of pH-responsive zwitterionic nanoparticles based on PLA/PEAd copolymers grafted with SBMA side chains. The copolymers were synthesized via a combination of ring-opening polymerization (ROP) and atom-transfer radical polymerization (ATRP) to finely control molecular architecture. The resulting zwitterionic copolymers were used to formulate paclitaxel (PTX)-loaded nanoparticles via a conventional oil-in-water (o/w) emulsification/solvent evaporation method. The influence of PEAd content on nanoparticle properties, including size, zeta potential, biodegradability, and drug release kinetics under acidic conditions, was investigated. Our findings demonstrate the potential of these zwitterionic PLA-based nanoparticles as efficient and biocompatible carriers for tumor-targeted chemotherapy, combining the structural tunability of biodegradable copolymers with the functional advantages of zwitterionic surface modification. Overall, this work provides evidence that SBMA-functionalized PLA/PEAd nanoparticles can effectively combine pH-responsiveness, biodegradability, and zwitterionic stealth characteristics, highlighting their potential in selective cancer therapy.

## 2. Materials and Methods

### 2.1. Materials

Ethylene glycol (ReagentPlus^®^, ≥99%, CAS Number: 107-21-1), adipic acid (ACS reagent, ≥99.0%, CAS Number: 124-04-9), and [2-(Methacryloyloxy)ethyl]dimethyl-(3-sulfopropyl)ammonium hydroxide (SBMA, 95%, CAS Number: 3637-26-1) were purchased from Sigma-Aldrich (Saint Louis, MO, USA). The catalysts and initiators used during synthesis (i.e., titanium(IV) butoxide (TBT, 97%, CAS Number: 5593-70-4), tin(II) 2-ethylhexanoate or stannous octoate (Sn(Oct)_2_, 92.5–100.0%, CAS Number: 301-10-0), α-Bromoisobutyryl bromide (BIBB, 98%, CAS Number: 20769-85-1), and 2,2′-Azobis(2-methylpropionitrile) (AIBN, 98%, CAS Number: 441090)), as well as poly(vinyl alcohol) (Mw 89,000–98,000, 99+% hydrolyzed, CAS Number: 9002-89-5), used as surfactant during nanoparticles preparation, were also supplied by Sigma-Aldrich. L-Lactide (98+%, CAS Number: 4511-42-6) was purchased from ThermoScientific Chemicals (Waltham, MA, USA). Paclitaxel (PTX, 99.5%, MW 853.91 g/mol, CAS Number: 33069-62-4), was purchased from Alfa Aesar (Stoughton, MA, USA). *Rhizopus delemar* and *Pseudomonas cepacia* lipases were purchased from Fluka BioChemika (Steinheim, Germany). All other reagents used were of either analytical or pharmaceutical grade.

### 2.2. Synthesis of Zwitterionic PLA/PEAd-SBMA Copolymers

#### 2.2.1. Synthesis of PEAd

**Scheme 1:**
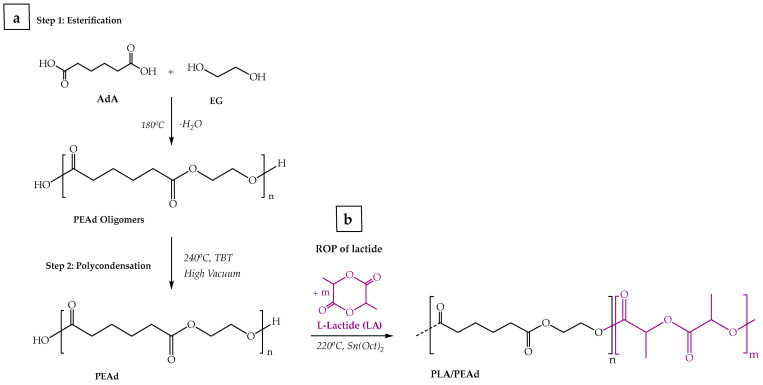
Schematic illustration of (**a**) the two-step poly(ethylene adipate) synthesis, and (**b**) the PLA/PEAd copolymers synthesis via ROP.

PEAd was prepared by the two-stage melt polycondensation method (esterification and polycondensation) in a glass batch reactor based on previously reported methods [19]. The two steps of the reaction are shown in Scheme 1a. In the first stage (esterification), PEAd oligomers were synthesized as follows; proper amounts of adipic acid and 1,2-ethyleneglycol in a 1/1.1 M ratio were placed in a round-bottom flask that was equipped with a mechanical stirrer, condenser and nitrogen inlet. This slight excess of glycol was selected to ensure complete consumption of the acid and to minimize the presence of residual carboxylic groups, which can reduce molecular weight and lead to premature chain termination. The polymerization mixture was de-gassed and purged with nitrogen several times, inserted into a heated salt bath at 180 °C and kept therein under constant stirring at 250 rpm, while gradually raising the reaction temperature up to 240 °C over a period of 4 h to balance esterification kinetics, while preventing uncontrolled foaming and side reactions. After complete distillation of the theoretical amount of water, the nitrogen flow was stopped, 400 ppm of TBT (0.05 g/mL in toluene) based on the reactants were added to the mixture and high vacuum (5.0 Pa) was slowly applied to avoid excessive foaming. The temperature was increased to 230 °C (400 rpm) and the polycondensation was carried out for 2 h. Both TBT concentration and the reaction temperature were selected according to the previous literature and our own optimization studies to ensure efficient polycondensation while avoiding discoloration or catalyst residues and to protect chain growth whilst avoiding the thermal degradation of PEAd, respectively.

#### 2.2.2. Synthesis of PLA/PEAd Copolymers

For the synthesis of PLA/PEAd copolymers were synthesized via ring opening polymerization (ROP) of L-lactide according to previously known protocols with some modifications (i.e., adjusting the reaction time and the catalyst concentration to control the resulting molecular weight distribution of the copolymers and to minimize the amount of unreacted lactide in the final product), were followed [38,39], and the synthetic route is depicted in Scheme 1b. In total, a set of three copolymers of varying L-lactide:PEAd weight ratios (95/5, 90/10 and 75/25 *w*/*w*) was produced. First, the reacting monomers, L-lactide and PEAd, were dried overnight at 50 °C under vacuum to remove moisture and then placed in a round-bottom reaction flask, adding stannous octoate (Sn(Oct)_2_) as the reaction catalyst (monomer-to-catalyst ratio of 400:1). The system was purged with nitrogen gas for 10 min to eliminate oxygen from the system, and the reaction proceeded in an oil bath at 220 °C under nitrogen and continuous stirring for 6 h. Upon completion, the reaction mixture was cooled in an ice bath, dissolved in dichloromethane (DCM), and precipitated in cold hexane. The precipitated copolymers were collected via vacuum filtration, washed with hexane, and dried in a vacuum oven at 50 °C for 24 h. The resulting copolymers were characterized by a combination of techniques discussed below and then stored in a desiccator at room temperature until further use. 

#### 2.2.3. Grafting of SBMA Monomer

**Scheme 2:**
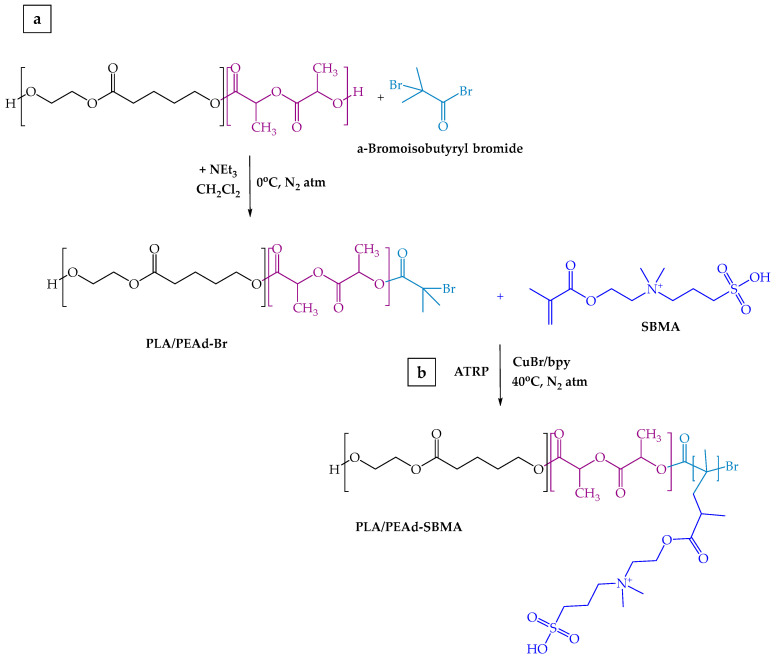
Schematic representation of two-step synthetic process of the zwitterionic PLA/PEAd-SBMA copolymers.

Drawing from previous literature reports [40], the grafting of the zwitterionic monomer [2-(Methacryloyloxy)ethyl]dimethyl-(3-sulfopropyl)ammonium hydroxide (SBMA) onto the PLA/PEAd copolymers was achieved via a two-step ATRP synthetic process using α-bromoisobutyryl bromide (BIBB) as the initiator (Scheme 2). First, an appropriate amount of PLA/PEAd copolymer was dissolved in anhydrous dichloromethane (DCM) and reacted with BIBB, in the presence of triethylamine (TEA) under nitrogen at RT for 18 h, to introduce bromide functional groups. The modified copolymer was then purified by precipitation in cold methanol and dried under vacuum. The ATRP reaction was carried out by dissolving the brominated PLA/PEAd in a mixture of SBMA monomer, CuBr catalyst, and 2,2′-bipyridine (bpy) ligand in a degassed methanol/water (1:1) solvent system. The reaction proceeded under nitrogen and continuous stirring at 40 °C for 24 h. Upon reaction completion, the zwitterionic copolymers were precipitated in an excess amount of acetone, filtered, dialyzed in a membrane tubing against deionized water for two days, and finally dried under vacuum. The SBMA-grafted copolymers were thoroughly characterized with a combination of supplementary techniques and then stored in a desiccator at room temperature until further use. 

### 2.3. Preparation of PTX-Loaded Nanoparticles

The zwitterionic nanoparticles were prepared using the oil-in-water (O/W) emulsification/solvent evaporation method using PVA as the surfactant. Briefly, 100 mg of each zwitterionic copolymer were dissolved in 5 mL of dichloromethane. Subsequently, 10 mg of the drug were added to the solution and homogeneously dispersed using an ultrasonic processor (UP100H, Hielscher, Teltow, Germany) at 100 watts and 30 kHz. An aqueous PVA solution (1% *w*/*v*) was also prepared, and 20 mL of it were added dropwise to the organic solution, followed by the emulsification of the two phases using again probe sonication for a total time of 2 min. The sonication parameters (time, intensity) were previously optimized to provide reproducible nanoparticles with uniform size distributions, and to ensure the protection of the active agent. The oil-in-water (o/w) emulsion was then mechanically stirred overnight to ensure complete solvent evaporation. NPs were collected by centrifugation at 11.000 rcf for 15 min, using the Heraeus Pico17 centrifuge (Thermo Electron Corporation, Waltham MA, USA). The experimental conditions were selected to ensure complete recovery of nanoparticles for all formulations, while minimizing particle aggregation and preventing the loss of smaller particles. The obtained NPs were washed twice with DI water, and the resulting aqueous nano-suspension was lyophilized using a Scavnac freeze-drier system (Coolsafe 110-4 Pro, LaboGene Scandinavia, Denmark) at −80 °C for 24 h, yielding the final dried NPs.

### 2.4. Characterization Methods 

#### 2.4.1. Attenuated Total Reflection Fourier Transform Infrared (ATR-FTIR) Spectroscopy

ATR-FTIR spectra were acquired using an IRTracer-100 spectrometer (Shimadzu, Kyoto, Japan), fitted with a QATR™ 10 single-reflection accessory and a diamond crystal. The spectra were collected over the range 450 to 4000 cm^−1^ at a resolution of 2 cm^−1^ (a total of 16 co-added scans), while the baseline was corrected and converted in-to absorbance mode.

#### 2.4.2. X-Ray Diffractometry (XRD)

X-ray powder diffraction (XRD) patterns were recorded using an XRD-diffractometer (Rigaku-Miniflex II, Tokyo, Japan) equipped with a CuKα radiation source (wavelength: 0.15405 nm). The instrument was operated at 30 kV and 15 mA, ensuring optimal resolution and intensity of the diffraction peaks. The samples were scanned over a 2θ range of 5° to 45° at a scanning rate of 1°/min with a step size of 0.05°.

#### 2.4.3. Differential Scanning Calorimetry (DSC)

DSC measurements were carried out using a Pyris Diamond DSC instrument (Perkin-Elmer, Dresden, Germany). Calibration was performed using zinc and indium standards. Nitrogen flow (50 mL/min) was applied to provide a constant thermal blanket within the DSC cell. Approximately 5 ± 0.1 mg of each sample was sealed in aluminum pans and subjected to heating 50 °C above its respective melting point at a rate of 20 °C/min to eliminate prior thermal history. Two subsequent heating scans of the quenched samples were recorded to observe the melting temperatures (T_m_). In the first scan, the samples were heated from 20 to 250 °C with a heating rate 20 °C/min, while in the second scan they were cooled to 0 ◦C with a cooling rate of 20 °C/min and heated again to 250 °C with a heating rate 20 °C/min.

#### 2.4.4. Thermogravimetric Analysis (TGA)

TGA measurements were carried out using a NETZSCH STA 449F5 instrument (NETZSCH Group, Germany) in the temperature range of 30–600 °C, with a heating rate of 20 °C min^−1^, under a nitrogen atmosphere.

#### 2.4.5. Gel Permeation Chromatoy (GPC)

The molecular weight of the materials was determined using Gel permeation chromatography (GPC) with a Waters 600 high-performance liquid chromatographic pump, Waters Ultrastyragel columns HR-1, HR-2, HR-4E, HR-4, and HR-5 (Waters Ultrastyragel, Milford, CT, USA), and a Shimadzu RID10A refractive index detector (Shimadzu RID10A, Kyoto, Japan). In total, 9 polystyrene (PS) standards of MW between 2.5 and 900 kg mol^−1^ were employed for the calibration. The prepared solutions had a 10 mg mL^−1^ concentration in chloroform; the injection volume was 150 mL, and the total elution time was 50 min. The oven temperature was 40 °C.

#### 2.4.6. In Vitro Enzymatic Degradation Testing

For the enzymatic degradation experiments, we employed *Rhizopus delemar* (0.09 mg/mL) and *Pseudomonas cepacia* (0.01 mg/mL) lipases, which are widely used in polyester degradation studies due to their strong ester hydrolytic activity, and for each material, thin films of same dimensions (1.5 cm × 1.5 cm) and thickness (~1 mm) were prepared, using an OttoWeber Type PW 30 hydraulic press (Paul-Otto Weber GmbH, Remshalden, Germany). Each specimen was placed in 10 mL phosphate-buffered saline of three different pH values (PBS, pH 7.4, 6.5, and 5.0). The specimens were stored in an incubator at 100 rpm, 37.0 ± 0.5 °C up to 30 days. The PBS solution was replaced weekly. All specimens were washed with distilled water to remove residual solution, and the wet weight of each specimen was measured. To measure the dry weight, specimens were dried in a vacuum oven for 48 h at 40 °C and then weighed. The difference between the initial mass (Wo) and the mass after the immersion (Wt) provided the initial mass of the degraded sample, thus the (%) weight loss of the sample was derived using the following equation:

(1) $$\%\;\mathrm{Mass}\;\mathrm{Loss}\;=\;\frac{Wo-Wt}{Wo}\;\times \;100.$$

#### 2.4.7. Water Contact Angle 

The water contact angle of all copolymers was measured using an Ossila L2004A1 contact angle goniometer (Ossila Ltd., Shiefield, UK) at 25 °C. The contact angle was determined by carefully placing a water droplet (5 μL) on the surface of the samples. All results were analyzed using the Ossila Contact Angle software 4.0.3.1.

#### 2.4.8. Particle Size and ζ-Potential Estimation (DLS)

The particle size and ζ-potential of the NPs were determined by dynamic light scattering (DLS), utilizing a Zetasizer Nano Instrument (Malvern Instruments, Nano ZS, ZEN3600, Malvern, UK) equipped with a 532 nm laser, using angle measurements of 90° at 25 °C. The samples were measured in suspension form, using an aqueous solution of NaCl (10^−4^ M) after sonication at 25 °C. For all samples, experiments were performed in triplicate and results are presented in mean values.

#### 2.4.9. Scanning Electron Microscopy (SEM)8

The surface morphology of films produced by the synthesized polymeric materials, as well as the size and morphology of the NPs were studied using scanning electron microscopy (SEM). Particularly, the samples were prepared with a thin layer of metal and covered with a carbon coating to provide a good conductivity of the electron beam before examining in a JEOL (JMS-840A) scanning microscope (Jeol Ltd., Akishima, Japan). SEM was performed with an accelerating voltage of 20 kV, probe current of 45 nA, and counting time of 60 s.

#### 2.4.10. Drug Loading and in Vitro Drug Release Studies

Paclitaxel’s loading and encapsulation efficiency (EE) was measured by dissolving 5.0 mg of NPs in dichloromethane and determining drug’s assay via HPLC (method details are given below). % Drug loading and EE values were calculated by using the following equations:

(2) $$\%\;\mathrm{Drug}\;\mathrm{loading}\;=\;\frac{weight\;of\;PTX\;in\;NPs}{weight\;of\;NPs}\;\times \;100,$$

(3) $$\%\;\mathrm{EE}=\frac{weight\;of\;PTX\;in\;NPs}{initial\;weight\;of\;PTX}\;\times \;100.$$

The dissolution experiments were carried out in a DISTEK Dissolution Apparatus I (Dissolution system 2100C, Distek, North Brunswick, NJ, USA) equipped with an autosampler (Evolution 4300, Distek, North Brunswick, NJ, USA) in 500 mL of aqueous phosphate buffer medium of three different pH values (pH 7.4, 6.5 and 5.0). The tested samples were placed in dialysis membrane tubing under sink conditions at 50 rpm and 37 °C. Two milliliters of aqueous solution were withdrawn from the release medium at predefined time intervals, and the PTX content was determined using a Shimadzu HPLC prominence system (Kyoto, Japan), equipped with a degasser (DGU-20A5, Kyoto, Japan), a liquid chromatograph (LC-20 AD, Kyoto, Japan), an autosampler (SIL-20AC, Kyoto, Japan), and a UV/Vis detector (SPD-20A, Kyoto, Japan). An Athena C18-WP (4.6 × 100 mm, 5 μm particle size) analytical column was used at a flow rate of 1.0 mL/min and 20 μL injection volume. The diode array detector was set at 227 nm, and the quantification of the API was based on a calibration curve previously prepared in the range of 0.1–50 μg/mL with r^2^ = 0.999 to mobile phase acetonitrile:water (70:30 *v*/*v*).

#### 2.4.11. Evaluation of Hemocompatibility

In vitro hemocompatibility of the studied copolymers, as well as the fabricated nanoparticle formulations, was assessed via a red blood cell (RBC) hemolysis assay. Freshly isolated erythrocytes were washed twice with phosphate-buffered saline (PBS, pH ~7.4), as previously described [41]. A 2% RBC suspension in PBS was prepared and incubated with nanoparticle suspensions (diluted in PBS) at final concentrations of 1, 0.5, 0.25, and 0.125 mg/mL in Eppendorf tubes. 

The same set of experiments was performed with the incubation of the RBCs suspension at 2% hematocrit in contact with the neat copolymers in film form. Accurately weighed samples were incubated at 37 °C for 24 h. Positive and negative controls were prepared using 2% RBCs lysed in distilled water and untreated RBCs in PBS, respectively. After incubation, tubes were centrifuged at 1600 rpm for 5 min and 100 μL of supernatant from each sample was transferred to a 96-well microplate for absorbance measurement at 540 nm. Hemolysis percentage was calculated using the formula:

(4) $$\%\;\mathrm{Hemolysis}\;=\;\frac{Abs\_sample\;-\;Abs\_negative}{Abs\_positive\;-\;Abs\_negative}\;\times \;100.$$

All measurements were performed in triplicate.

#### 2.4.12. In Vitro Cytotoxicity and Cell Viability

Cells from the MDA-MB-231 human breast cancer line and immortalized mouse embryonic fibroblasts (iMEFs) were seeded in both 12-well and 96-well plates and cultured for 24 h in standard growth medium to allow for attachment and recovery. Conditioned media were prepared by incubating each NPs compound in complete medium at a concentration of 1 mg/mL for 5 days. For all cytotoxicity experiments, the effective paclitaxel concentration in the nanoparticle extracts was normalized based on the drug loading and encapsulation efficiency (EE) of each formulation to ensure comparable PTX doses across all samples. For the treatments, NP solutions were diluted to 1:5 or 1:20 (*w*/*v*) and replaced the growth medium in the cell cultures. Incubation with the treatments lasted for 18 h. Cell proliferation and viability were assessed through fluorescent microscopy and nuclear staining (Hoechst) to evaluate morphological changes and adherence. CellTox™ Green (Promega) membrane integrity staining was also used to evaluate late apoptosis. Hoechst and CellTox signals were imaged by fluorescence microscopy and used to quantify total cell number and cytotoxicity, respectively. 

## 3. Results and Discussion

### 3.1. Characterization of the Zwitterionic Copolymers

#### 3.1.1. Structural Characterization (ATR-FTIR, GPC)

**Figure 1:**
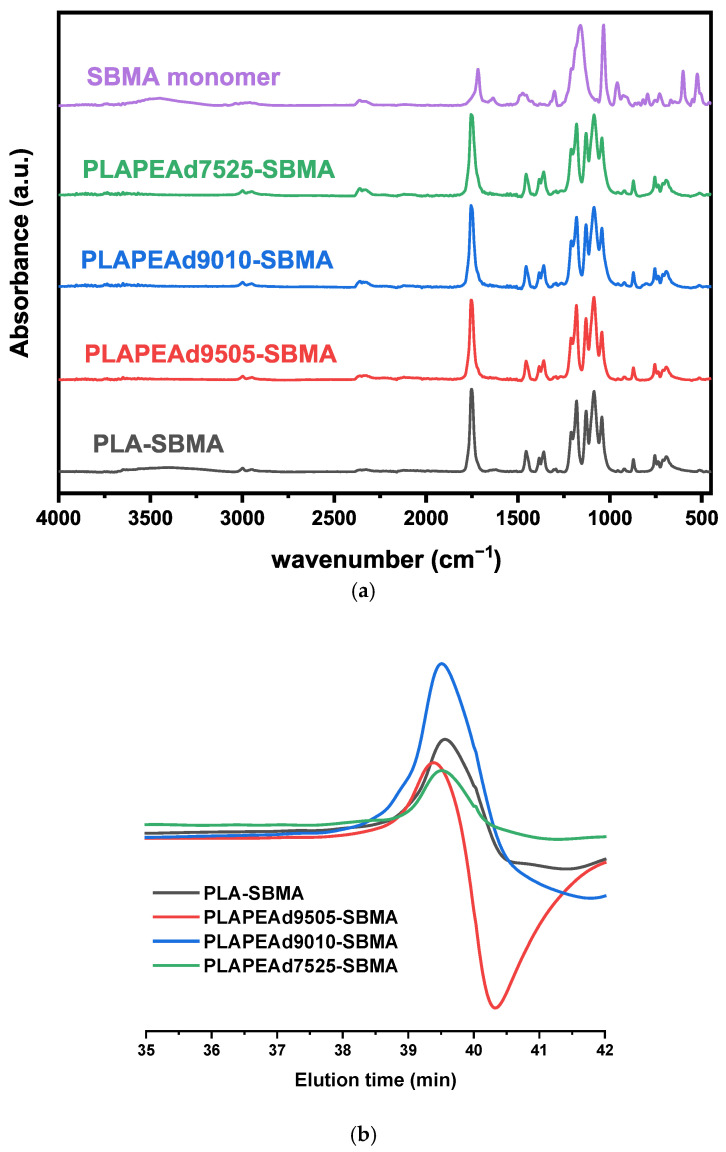
(**a**) FTIR spectra confirming SBMA grafting onto PLA and PLA/PEAd copolymers. (**b**) GPC chromatograms showing molecular weight distributions of synthesized zwitterionic copolymers.

Fourier-transform infrared (ATR-FTIR) spectroscopy was employed to confirm the successful grafting of the SBMA monomer upon the PLA and PLA/PEAd polymer backbone (Figure 1a). The characteristic ester bond signals of the polyester were clearly observed in all SBMA-grafted copolymers spectra, with the intense C=O stretching vibration around 1720 cm^−1^ and the C–O–C asymmetric stretching vibrations close to 1080 cm^−1^. The successful introduction of the zwitterionic moieties was evidenced by the appearance of new absorption bands at ~1030 cm^−1^, attributed to the symmetric stretching of sulfonate (SO_3_^−^) groups, and a shoulder around 1150 cm^−1^, representing the S=O asymmetric stretching of the sulfonate group. On the other hand, the broad band at ~3350 cm^−1^ corresponding to the overlapping O–H and N–H stretching vibrations confirmed the presence of hydrophilic SBMA side chains. The methyl and methylene C–H stretching vibrations appeared around 2870 cm^−1^, while minor bands near 1645 cm^−1^ and 1180–1175 cm^−1^ were also detected, corresponding to amide I regions and further contributions from S–O vibrations, respectively. In general, comparison between neat PLA/PEAd copolymers (Supplementary Figure S1) and their SBMA-grafted counterparts highlighted the formation of additional functional groups indicative of successful copolymer modification, especially in the region of 1030–1080 cm^−1^ and 1150 cm^−1^, whilst no significant shift in the main ester-related peaks was observed post-grafting, indicating that the backbone polymer architecture remained intact and the SBMA modification occurred primarily through side-chain functionalization. 

Gel permeation chromatography (GPC) was used to evaluate the molecular weight characteristics of the synthesized zwitterionic copolymers. All samples exhibited monomodal chromatographic profiles, indicative of well-controlled polymerization without evidence of significant crosslinking or multimodal populations (Figure 1b). The number-average molecular weights (Mn) ranged from 3200 to 4900 g/mol, and the weight-average molecular weights (Mw) from 3800 to 6700 g/mol (Table 1). PLA-SBMA showed a sharp, symmetrical peak corresponding to a number-average molecular weight (Mn) of 4400 g/mol and a narrow polydispersity index (PDI) of 1.28. PLAPEAd9010-SBMA, with the lowest Mn (3200 g/mol) and PDI (1.17), demonstrated the highest uniformity among all formulations. In contrast, PLAPEAd7525-SBMA exhibited a broader peak and a tailing pattern, reflected in its higher PDI of 1.48, consistent with the greater heterogeneity introduced by the higher PEAd content. Notably, the PLAPEAd9505-SBMA copolymer (red trace in Figure 1b) showed a symmetric primary peak and an intermediate molecular weight (Mn = 4900 g/mol, PDI = 1.38), as well as a subtle broad signal at higher retention times, suggesting the presence of a small fraction of low-molecular-weight species—possibly unreacted monomers or short-chain oligomers. Although we did not perform additional analyses within the context of this study, such as MALDI-TOF MS or LC–MS, that would allow the identification of these low-molecular-weight species, the proportion of this fraction, based on our existing data, is small and is unlikely to significantly alter the bulk polymer properties.

Compared to the neat PLA and PLA/PEAd copolymers, the zwitterionic SBMA-grafted counterparts exhibited significantly lower molecular weights and narrower molecular weight distributions, attributed to the ATRP grafting process, which introduces radical grafting sites that limit chain extension due to steric hindrance and termination, thus restricting molecular weight, but improving control over dispersity. Similar behavior has been reported for PLA-based graft copolymers, where controlled radical routes yield narrower dispersities at the expense of chain length [42]. As shown in the Supplementary GPC data (Table S1), neat PLA displayed a high Mn of 42,264 g/mol and Mw of 54,090 g/mol (PDI = 1.30), while the PLAPEAd9505, PLAPEAd9010, and PLAPEAd7525 copolymers exhibited decreasing Mn values of 39,000, 34,600, and 22,700 g/mol, respectively, with PDIs ranging from 1.4 to 1.5. In contrast, the corresponding zwitterionic samples recorded Mn values in the range of 3200–4900 g/mol and much lower PDIs (1.17–1.48), indicating a drastic reduction in polymer chain length, but also improved control over molecular weight distribution. This reduction is attributed to the distinct two-step polymerization strategy employed for the zwitterionic copolymers, particularly the ATRP-based SBMA grafting step, which inherently limits chain extension due to steric effects and radical termination events [43]. 

#### 3.1.2. Crystallinity and Thermal Properties (XRD, DSC, TGA)

**Figure 2:**
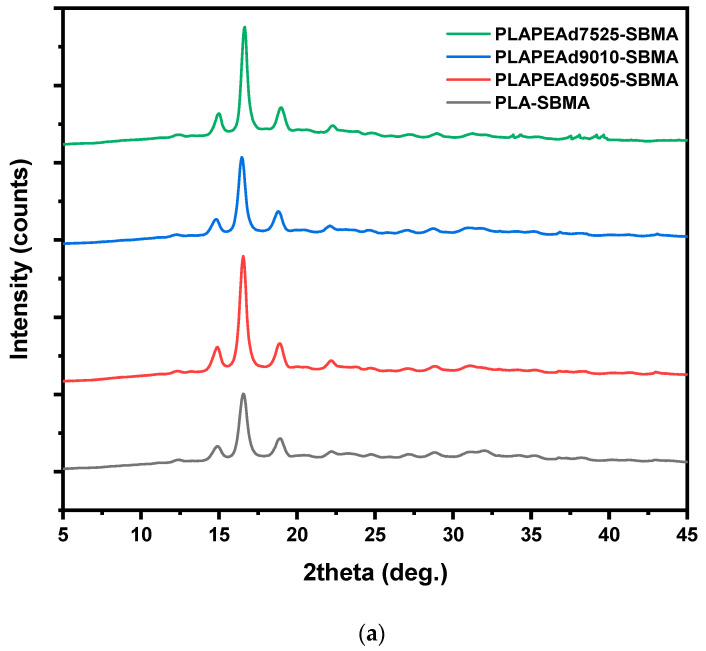

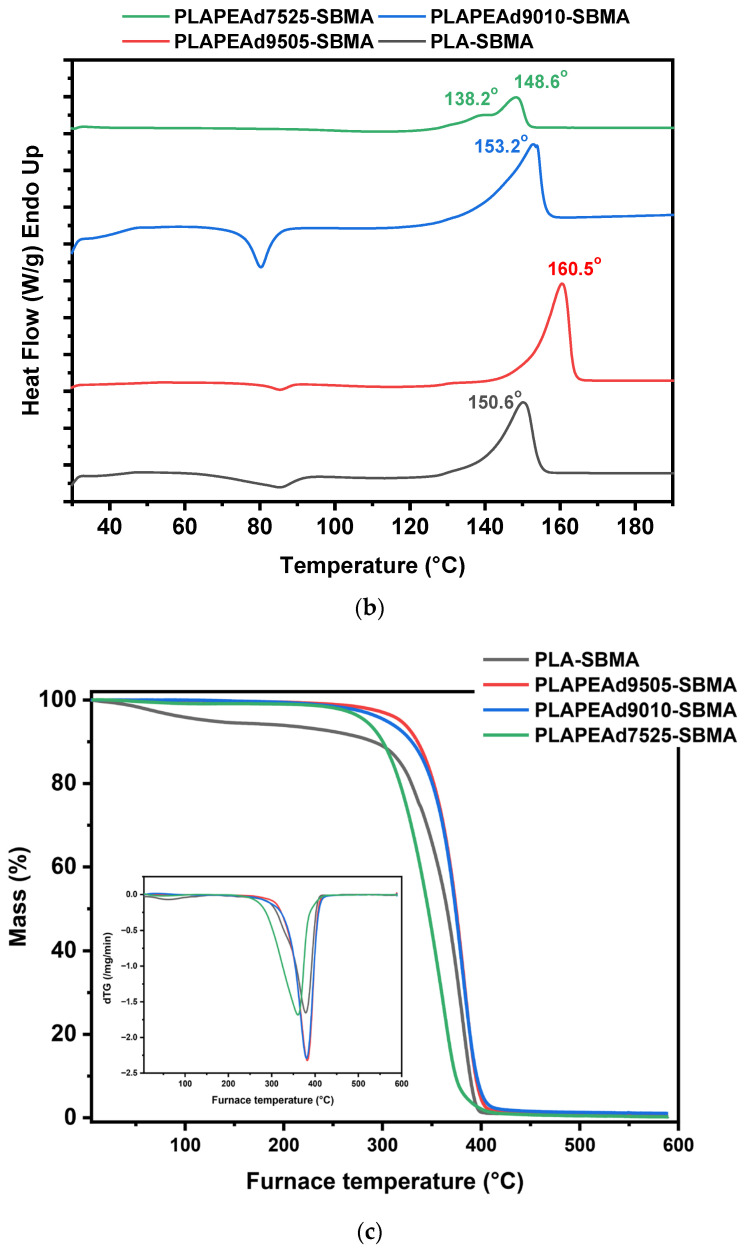
(**a**) XRD diffractograms, (**b**) DSC thermograms, and (**c**) TGA curves of the synthesized PLA-SBMA and PLA/PEAd-SBMA copolymers.

To better understand how the material composition affects chain mobility, thermal stability and molecular packing, and consequently the nanoparticle formation, their degradation behavior and thus drug release kinetics, it is essential to assess their crystallinity and thermal behavior. Therefore, a thorough investigation by means of X-ray diffraction (XRD), differential scanning calorimetry (DSC), and thermogravimetric analysis (TGA) was carried ouy, as presented in Figure 2.

X-ray diffraction (XRD) analysis was employed to assess the crystalline structure of the zwitterionic copolymers and how it is influenced by PEAd content and SBMA grafting (Figure 2a). All SBMA-grafted samples displayed a semicrystalline character, evidenced by three distinct diffraction peaks located at 2θ ≈ 14.8°, 16.6°, and 18.8°, which are attributed to the (010), (110)/(200), and (203) lattice planes of the orthorhombic α-form crystalline phase of PLA, respectively [44]. Notably, the intensity and sharpness of these peaks decreased progressively with increasing PEAd content, reflecting a reduction in overall crystallinity. This trend was most pronounced in PLAPEAd7525-SBMA, where the broadening of all three peaks suggests significant disruption of PLA crystalline domains due to the flexible, amorphous nature of the PEAd segments. These results align with Klonos et al. [20], who demonstrated that in PLA/PEAd blends, PEAd acts as a plasticizer that lowers PLA’s Tg, enhances segmental dynamics, and enables microphase-separated structures with tailored crystallization behavior.

DSC analysis (Figure 2b) supported these structural observations. PLA-SBMA exhibited a melting temperature (Tm) of ~151 °C, consistent with semicrystalline PLA. Increasing the PEAd content led to a systematic decrease in melting enthalpy (ΔHm), indicative of reduced crystallinity. As expected, PLAPEAd7525-SBMA presented the lowest ΔHm, confirming the significant disruption of ordered domains. However, a notable exception was observed in PLAPEAd9505-SBMA, which exhibited a higher Tm (~160 °C) compared to the rest. This differentiation in thermal behavior likely stems from its higher molecular weight (Mn = 4900 g/mol) and broader molecular weight distribution (PDI = 1.38), as confirmed by GPC. Longer chain lengths may enhance segmental interactions and provide greater potential for chain folding, which facilitates lamellar thickening and the formation of more stable crystalline regions during thermal treatment. Furthermore, the broader dispersity could further contribute to this effect by allowing a subset of longer chains to act as nucleation centers, promoting heterogeneous crystallization. These two processes combined result in crystalline domains with higher thermal stability, thereby shifting the Tm upward despite the presence of flexible PEAd segments.

Moreover, a distinct double melting endotherm was observed in the DSC thermogram of PLAPEAd7525-SBMA, indicating the presence of heterogeneous crystalline domains and possibly occurring melting–recrystallization–remelting phenomena. The high PEAd content (25 wt%) introduces extensive chain flexibility to the system and disrupts the regularity of the PLA crystalline lattice, leading to crystallite heterogeneity and irregular crystal formation. Thus, upon heating, the less stable and thinner crystallites melt first, which then allows polymer segments to reorganize into more stable lamellae that subsequently remelt at higher temperatures, resulting in a bimodal endothermic profile. This process is consistent with the broader molecular weight distribution (PDI = 1.48) of the sample, that indicates a wide range of chain lengths and contributes to the coexistence of crystals of varying stability.

TGA results (Figure 2c) revealed single-step thermal degradation for all copolymers, confirming their homogeneity. The thermal stability decreased slightly with increasing PEAd content. PLA-SBMA showed an initial degradation temperature (Tonset) around 258 °C, whereas PLAPEAd7525-SBMA began degrading near 243 °C. This decline is attributed to the incorporation of the less thermally stable PEAd soft segment. Despite this, all materials remained stable up to ~240 °C, which is sufficient for typical solvent-based processing techniques for nanoparticles fabrication. The absence of multiple degradation steps suggests uniform incorporation of the PEAd and SBMA moieties without phase separation. Comparing these observations with the TGA curves of the neat copolymers presented in Supplementary Figure S2, the influence of SBMA grafting on thermal behavior is evident. The neat PLA and PLA/PEAd copolymers exhibited slightly higher degradation onset temperatures, at 260–270 °C, which reflects the absence of the thermally less stable SBMA side chains. The incorporation of SBMA introduces sulfonate and quaternary ammonium functionalities that are known to degrade at lower temperatures, thereby shifting Tonset downward. In this context, the observed decrease in thermal stability is primarily attributed to zwitterionic functionalization rather than PEAd content.

#### 3.1.3. In Vitro Degradation Under Physiological and Acidic Conditions

**Figure 3:**
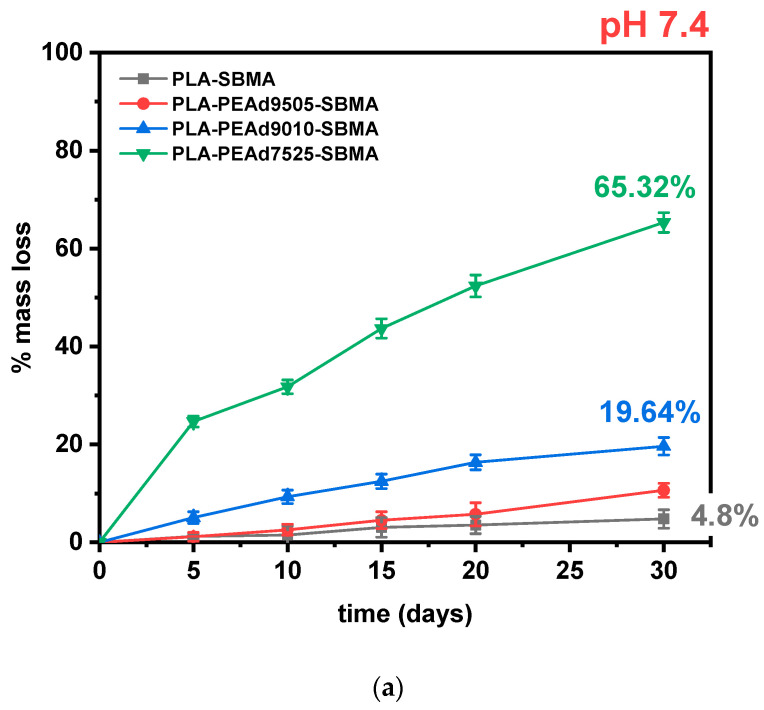

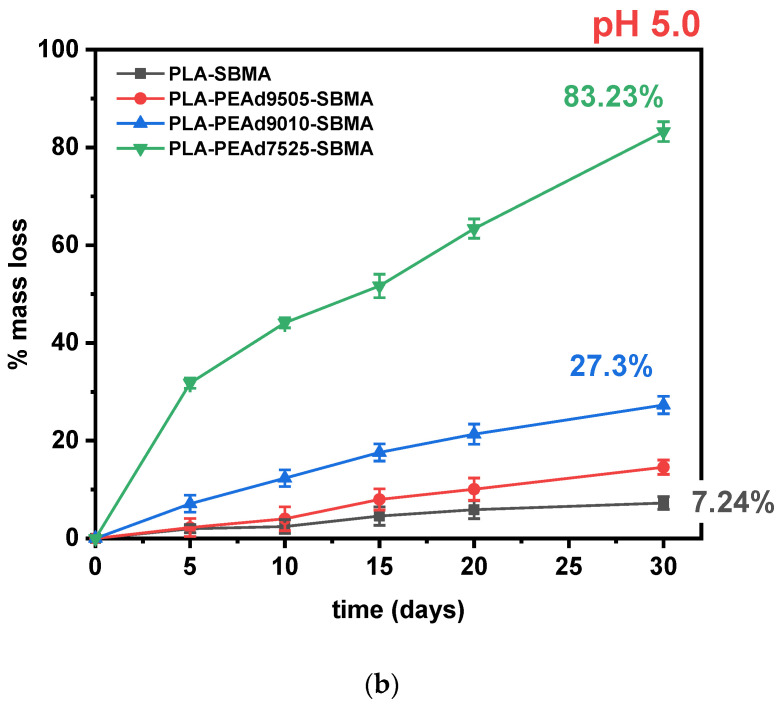
(**a**) Mass loss (%) of zwitterionic copolymers during in vitro degradation at pH 7.4 over 30 days. (**b**) Comparative degradation kinetics at pH 5.0, highlighting pH sensitivity. All data points up to 30 days are shown.

To assess the pH-responsive degradation behavior of the synthesized zwitterionic copolymers, hydrolytic degradation studies were conducted under simulated physiological (pH 7.4) and tumor-simulating acidic (pH 5.0) conditions over a 30-day period (Figure 3). The mass loss profiles demonstrated a clear dependence on both pH environment and copolymer composition. As expected, PLA-SBMA exhibited minimal degradation under neutral conditions, with less than 10% weight loss at pH 7.4, attributed to its semicrystalline structure and low water permeability. Its degradation rate remained low even under acidic conditions, confirming the inherent hydrolytic resistance and limited water penetration of the PLA matrix. In contrast, the incorporation of PEAd segments significantly increased degradation susceptibility, especially under acidic conditions. PLAPEAd7525-SBMA, which contains the highest PEAd content and lowest crystallinity (as evidenced by XRD and DSC), underwent rapid degradation, reaching 65% and 83% mass loss at pH 7.4 and 5.0, respectively. Similarly, PLAPEAd9010-SBMA and PLAPEAd9505-SBMA exhibited intermediate degradation rates, correlating with their moderate PEAd content and thermal/molecular properties. Mechanistically, under acidic conditions, ester hydrolysis proceeds via protonation of the carbonyl oxygen, increasing electrophilicity and accelerating chain scission; in aliphatic polyesters, this is further amplified by autocatalysis, as carboxylic acid end-groups formed during cleavage locally lower the microenvironmental pH and promote bulk erosion rather than purely surface-limited hydrolysis. This pathway is well documented for PLA-based materials and related polyesters [13,45]. 

In our copolymers, increasing the amorphous fraction (via higher PEAd content) raises water uptake and diffusivity, making ester bonds more accessible to protons and water, therefore hydrolytic cleavage occurs preferentially in the amorphous domains, consistent with classic observations for PLA and other semicrystalline polyesters [46]. Moreover, the PEAd blocks contribute a higher density of more labile aliphatic diester linkages and greater chain mobility, which are known to hydrolyze more readily than the tighter, more crystalline lactide sequences; studies on adipate-containing polyesters (e.g., PBAT) show the adipate-containing ester sites are primary hydrolysis loci, supporting our interpretation for PEAd-rich segments [47]. The expected pH sensitivity of the zwitterionic samples is confirmed in Figure 3b with faster and more extensive degradation occurring at lower pH values. 

**Figure 4:**
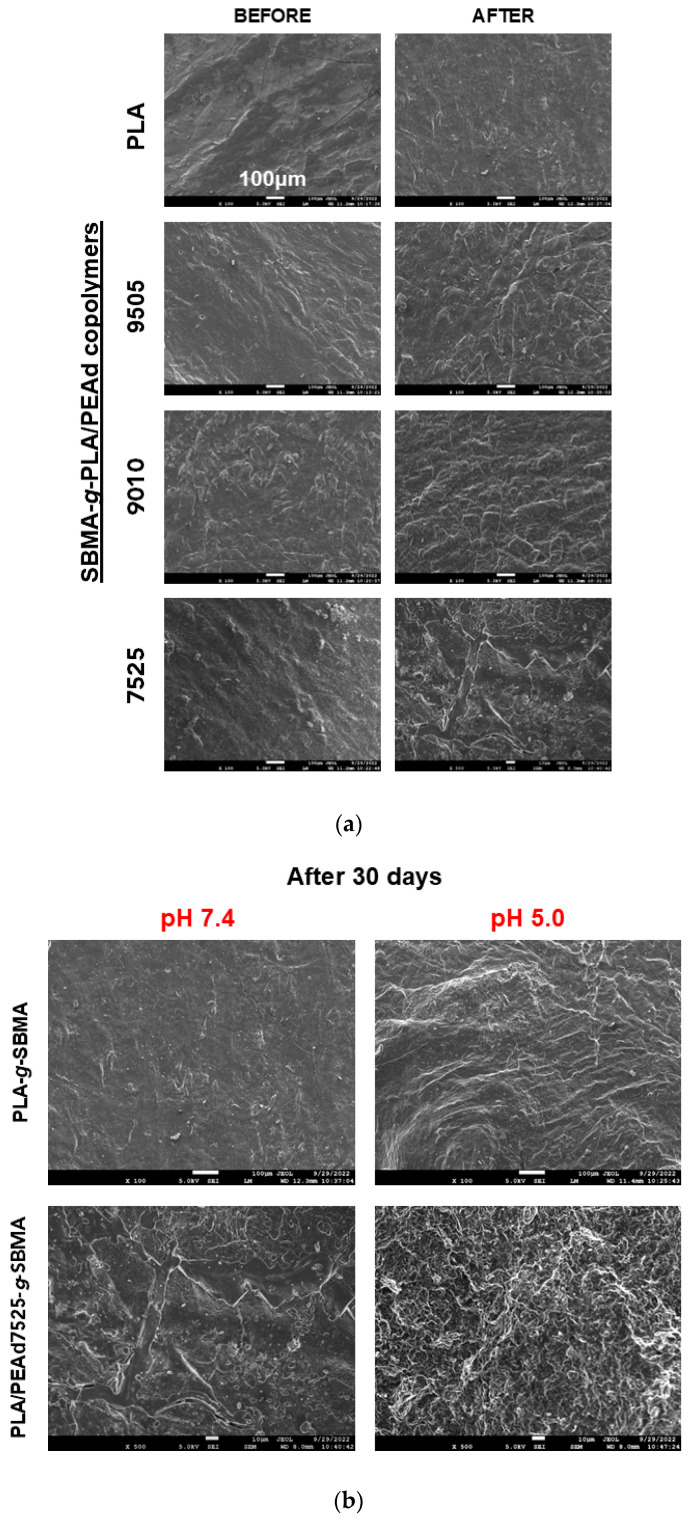
SEM images demonstrating surface morphology changes in copolymer films during degradation (**a**) over 30 days of study, and (**b**) in acidic PBS, supporting accelerated hydrolysis at pH 5.0.

SEM analysis of the copolymer films (Figure 4a) revealed distinct surface morphological changes correlated with copolymer composition and environmental pH. Initially, all films exhibited smooth and continuous surfaces, indicative of homogenous copolymer structure. Upon 30 days of incubation in phosphate-buffered saline (PBS), significant differences emerged among the samples. PLA-SBMA films retained a relatively smooth surface with minor surface pitting, consistent with the slow and surface-limited hydrolysis typically observed in semicrystalline, hydrophobic polyesters. In contrast, the PLA/PEAd-SBMA copolymers, particularly PLAPEAd7525-SBMA, showed extensive surface erosion characterized by deep cracks, pores, and fragment detachment. These features suggest a shift toward bulk erosion, driven by increased water permeability and reduced crystallinity due to the higher PEAd content. The incorporation of flexible PEAd blocks lowers crystallinity, as confirmed by XRD and DSC data in our study, creating a larger amorphous fraction that is more easily penetrated by water molecules. This disrupted packing in PEAd-rich copolymers allows water to permeate more uniformly, promoting chain scission throughout the matrix, compared to semicrystalline PLA, where crystalline domains typically act as barriers to water uptake, confining hydrolysis to the surface.

As previously discussed, the matrix degradation was further accelerated and intensified under acidic conditions. Figure 4b visually confirms this extended loss of surface integrity and more pronounced porosity, in the case of PLA-SBMA and PLAPEAd7525-SBMA films, depicting the minimum and maximum effect, respectively. As can be seen, the microstructural deterioration correlates well with the quantitative mass loss data and underlines the crucial role of the PEAd segment in modulating degradation kinetics, further validating the potential of these materials for stimuli-responsive drug delivery applications. It is important to mention that in our study, we used copolymer films to provide controlled, reproducible conditions and to allow correlation with thermal, crystallinity, and morphology data. While degradation rates may differ in nanoparticles due to their substantially higher surface area-to-volume ratio, which enhances water uptake and accelerates hydrolytic scission, the composition-dependent trends (e.g., faster degradation of PEAd-rich formulations and enhanced sensitivity under acidic pH) are expected to remain consistent.

#### 3.1.4. Wettability Measurements

**Figure 5:**
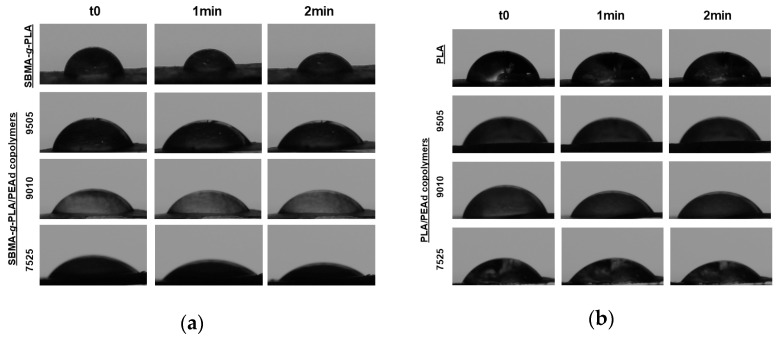
Static water contact angle images for (**a**) zwitterionic copolymer films and (**b**) neat copolymer films, demonstrating enhanced hydrophilicity with increased PEAd content and upon SBMA grafting. In both panels, images are shown from top to bottom for PLA, PLAPEAd9505, PLAPEAd9010, and PLAPEAd7525. For each sample, contact angles are displayed at three time points (0, 1, and 2 min).

Contact angle measurements demonstrated that SBMA grafting substantially enhanced the surface hydrophilicity of both neat PLA and PLA/PEAd copolymers (Figure 5). The static water contact angle of PLA-SBMA was measured at approximately 78°, significantly lower than that of unmodified neat PLA, which exhibited angles above 95° in prior studies. This improvement can be attributed to the zwitterionic nature of SBMA, which introduces ionic functionalities and increases surface polarity [48], which results in enhanced water affinity, thus better hydration and reduced interfacial tension. The incorporation of PEAd further modulated the surface wettability in a composition-dependent manner. This progressive reduction is consistent with the increasing content of soft PEAd, that introduces amorphous character and additional ester linkages capable of interacting with water molecules. Most importantly, these observations contrast with their neat PLA/PEAd counterparts, which generally showed larger contact (Figure 5b), reflecting their lower surface polarity and semi-hydrophobic nature prior to SBMA grafting. The observed trend aligns with the copolymers’ degradation behavior, as enhanced surface wettability is known to facilitate water uptake, thus accelerating hydrolytic degradation [49]. Furthermore, increased hydrophilicity can improve the colloidal stability of nanoparticles in aqueous media and reduce nonspecific protein adsorption, factors being both critical for stealth behavior in systemic circulation [50]. Therefore, the combined effect of SBMA grafting and PEAd incorporation allows for tunable control of surface hydration and wettability, improving both the processing characteristics and biological performance of the material.

### 3.2. Characterization of the PTX-Loaded Zwitterionic Nanoparticles

#### 3.2.1. Structural and Morphological Characterization (FTIR, SEM)

**Figure 6:**
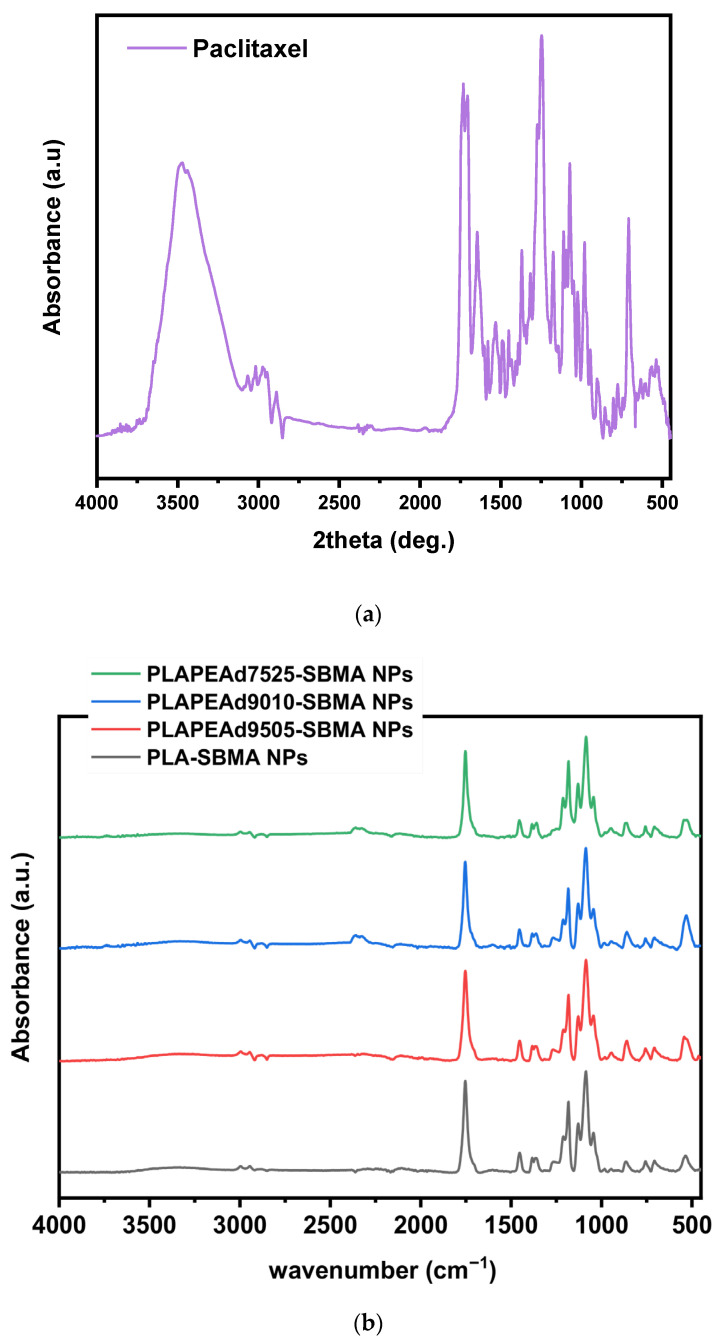
FTIR spectra of (**a**) pure PTX, and (**b**) the prepared PTX-loaded nanoparticles.

To confirm the presence of paclitaxel (PTX) within the zwitterionic nanoparticles and to assess potential drug–polymer interactions, FTIR analysis was employed. Figure 6a demonstrates the FTIR spectrum of pure PTX, exhibiting multiple characteristic bands attributed to its complex molecular structure, including a broad absorption band in the 3600–3400 cm^−1^ region corresponding to O–H and N–H stretching vibrations from hydroxyl and amide groups, several peaks in the 2950–2850 cm^−1^ region arising from aliphatic C–H stretching vibrations, the ester carbonyl (C=O) stretching vibration at 1731 cm^−1^, aromatic ring C=C stretches between 1600 and 1450 cm^−1^, amide-related vibrations near 1650–1630 cm^−1^, and absorption peaks in the 1240–1050 cm^−1^ range due to C–O–C ether stretches as well as secondary alcohols. Finally, the absorption band around 530 cm^−1^ corresponds to C–C skeletal bending vibrations or ring deformation modes, which are particularly typical to aromatic or fused ring systems, such as the taxane core present in PTX.

In the FTIR spectra of the drug-loaded nanoparticles (Figure 6b), the previously discussed characteristic absorption bands of the copolymer matrix remained dominant. Specifically, the ester carbonyl peak appears at 1720 cm^−1^, C–O–C stretch near 1080 cm^−1^, and SO_3_^−^ sulfonate band at ~1030 cm^−1^. Most of the characteristic peaks associated with pure PTX, particularly in the 1600–1500 cm^−1^ aromatic region and the 1240–1050 cm^−1^ ether region, were either absent or significantly weak, most likely due to overlapping with the signals arising from the polymeric structure and the low drug amount within the polymeric phase. However, the appearance of a new low-frequency absorption band at 530 cm^−1^, which was not present in the spectra of the neat copolymers, can be attributed to the out-of-plane bending or ring deformation modes of PTX. Moreover, the complete absence of any band between 3600 and 3400 cm^−1^ (O–H, N–H), suggests that PTX’s hydrogen bonding environment was altered, likely due to the homogeneous distribution within the matrix or reduction in free hydroxyl availability due to interactions with the copolymer, whereas slight peak broadening and subtle shifts (e.g., around 1720–1730 cm^−1^) further suggest weak intermolecular interactions between PTX and the polar groups of the zwitterionic copolymer (e.g., ester or sulfonate moieties). 

**Figure 7:**
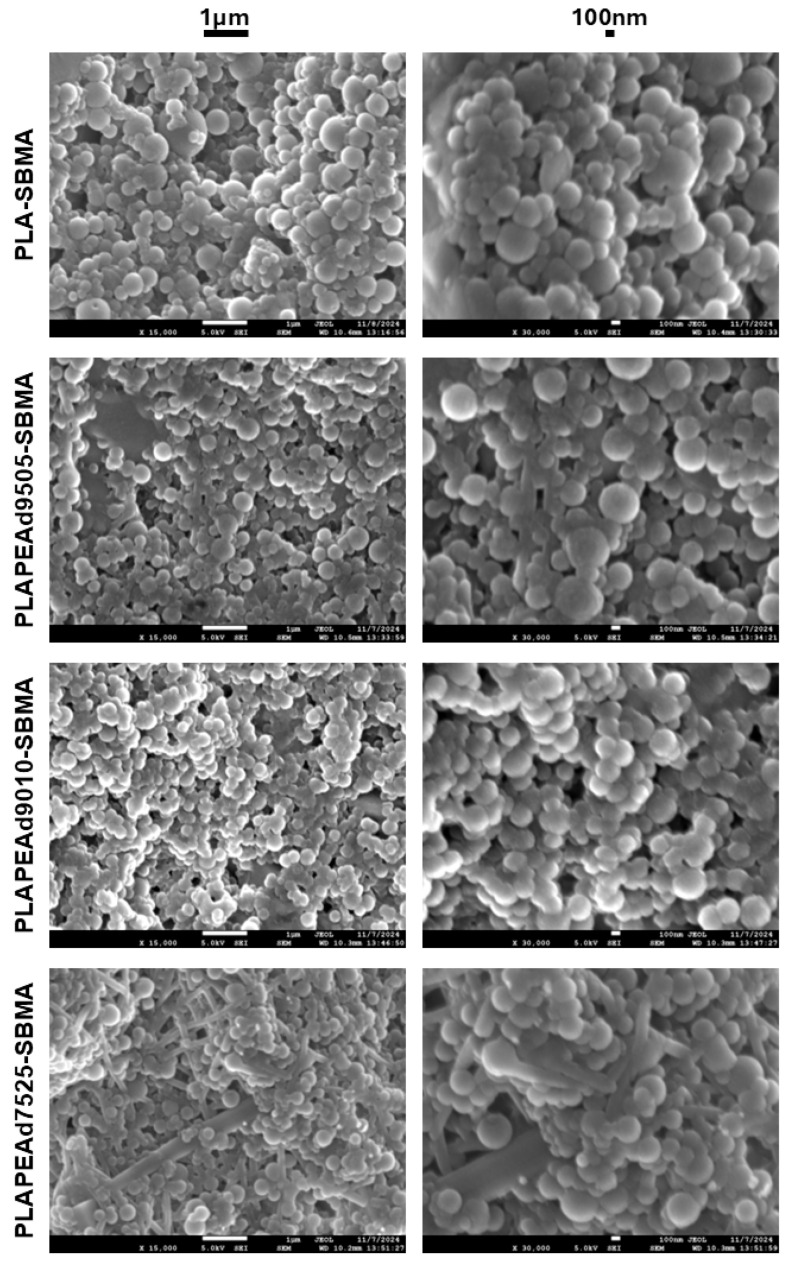
SEM images of two different magnifications, revealing morphology and surface characteristics of the fabricated nanoparticles.

SEM images confirmed the successful formation of paclitaxel-loaded nanoparticles with predominantly spherical morphology and smooth surface texture (Figure 7). Across all formulations, the emulsification/solvent evaporation technique yielded discrete particles without visible cracks or surface defects. Differences in nanoparticle size distribution and surface features were observed depending on the copolymer composition. Nanoparticles based on PLA-SBMA appeared to be the smallest and most uniform, with diameters below 250 nm and highly defined contours. This observation is consistent with the higher crystallinity, greater chain rigidity, and narrower molecular weight distribution (PDI = 1.28) of the copolymer, which support tighter packing and better shape retention during the solvent evaporation phase. PLAPEAd9010-SBMA nanoparticles appeared larger and less compact than PLA-SBMA-based particles, though still well-formed and discrete, reflecting a balance between chain flexibility and stabilization. Interestingly, PLAPEAd9505-SBMA nanoparticles exhibited a favorable compromise: they retained spherical morphology and smooth surfaces, with slightly larger but uniform diameters compared to PLA-SBMA. These observations match well with their intermediate PEAd content and relatively high Mn (4900 g/mol), which may improve chain entanglement and interfacial stabilization during nanoprecipitation.

Among all formulations, the PLAPEAd7525-SBMA nanoparticles exhibited the most heterogeneous morphology, with the presence of not only spherical particles but also irregular rod-like or fibrillar structures visible on the surface, which however represented only a minor population. These rod-like features are likely a consequence of the high PEAd content (25 wt%), which introduces extensive chain flexibility and reduces crystallinity, leading to phase instability during emulsification and solidification. The increased segmental mobility, coupled with the broad polydispersity (PDI = 1.48), may result in partial phase separation or anisotropic chain packing, especially during solvent evaporation. Additionally, the reduced viscosity of the organic phase associated with the low molecular weight and soft-segment-rich composition may favor elongated micelle-like aggregation or chain coalescence into rod-like structures, rather than uniform spherical droplet formation. 

#### 3.2.2. Particle Size Analysis

**Figure 8:**
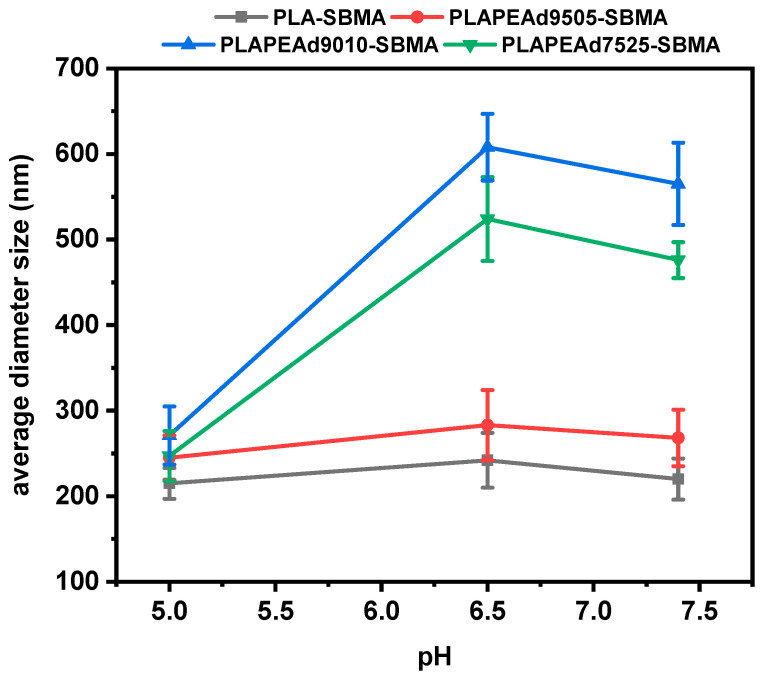
DLS particle size analysis of PTX-loaded nanoparticles under different pH conditions.

Dynamic light scattering (DLS) measurements confirmed the successful fabrication of paclitaxel-loaded nanoparticles with hydrodynamic diameters ranging from 220 to 565 nm at pH 7.4 (Table 2, Figure 8). The smallest nanoparticles were obtained from PLA-SBMA, with an average size of 220 ± 24 nm, consistent with this copolymer’s higher crystallinity, narrower molecular weight distribution (PDI = 1.28), and more rigid chain structure—factors that promote compact packing during nanoparticle formation. As PEAd content increased, particle sizes became progressively larger. PLAPEAd9010-SBMA and PLAPEAd7525-SBMA nanoparticles showed notably larger hydrodynamic diameters (565 ± 48 nm and 476 ± 29 nm, respectively), reflecting the influence of the soft, amorphous PEAd segments that introduce greater chain mobility, lower packing density, and higher swelling potential. PLAPEAd9505-SBMA exhibited intermediate behavior, forming nanoparticles of 268 ± 33 nm, with relatively low polydispersity and good morphological uniformity. This aligns with its favorable combination of moderate PEAd content, relatively high molecular weight (Mn = 4900 g/mol), and good thermal characteristics, all of which contribute to better nanoparticle stability and reduced aggregation, as previously observed in SEM analysis.

A key advantage of the zwitterionic nanoparticles was their pronounced pH-responsive size modulation. At acidic pH values (6.5 and especially 5.0), all nanoparticle formulations exhibited significant reductions in hydrodynamic diameter, a desirable trait for tumor-targeted delivery. This effect was particularly evident in PLAPEAd9010-SBMA, where particle size decreased from 565 nm to 271 nm, and in PLAPEAd7525-SBMA, from 476 nm to 247 nm. Such shrinkage is likely driven by acid-catalyzed hydrolysis of ester bonds, enhanced polymer degradation, and subsequent collapse or compaction of the polymer network. The effect was less pronounced in PLA-SBMA, whose semicrystalline, hydrolytically resistant structure limited pH responsiveness. This pH-dependent size shrinkage supports the copolymers’ degradation data (Figure 3) and confirms the dual contribution of PEAd content and zwitterionic character to the environmental responsiveness of the system. Moreover, the presence of zwitterionic SBMA side chains contributes not only to the observed pH-responsive size modulation, but also to improved colloidal stability through enhanced hydration and charge shielding. This dual function, recently reviewed in detail [51], is also consistent with our findings, where all formulations maintained stable colloidal dispersions across physiological and acidic pH conditions. In overall, the reversible size changes could facilitate enhanced tumor penetration, cellular uptake, and controlled drug release specifically in acidic microenvironments.

#### 3.2.3. Crystalline Structure and Thermal Analysis

**Figure 9:**
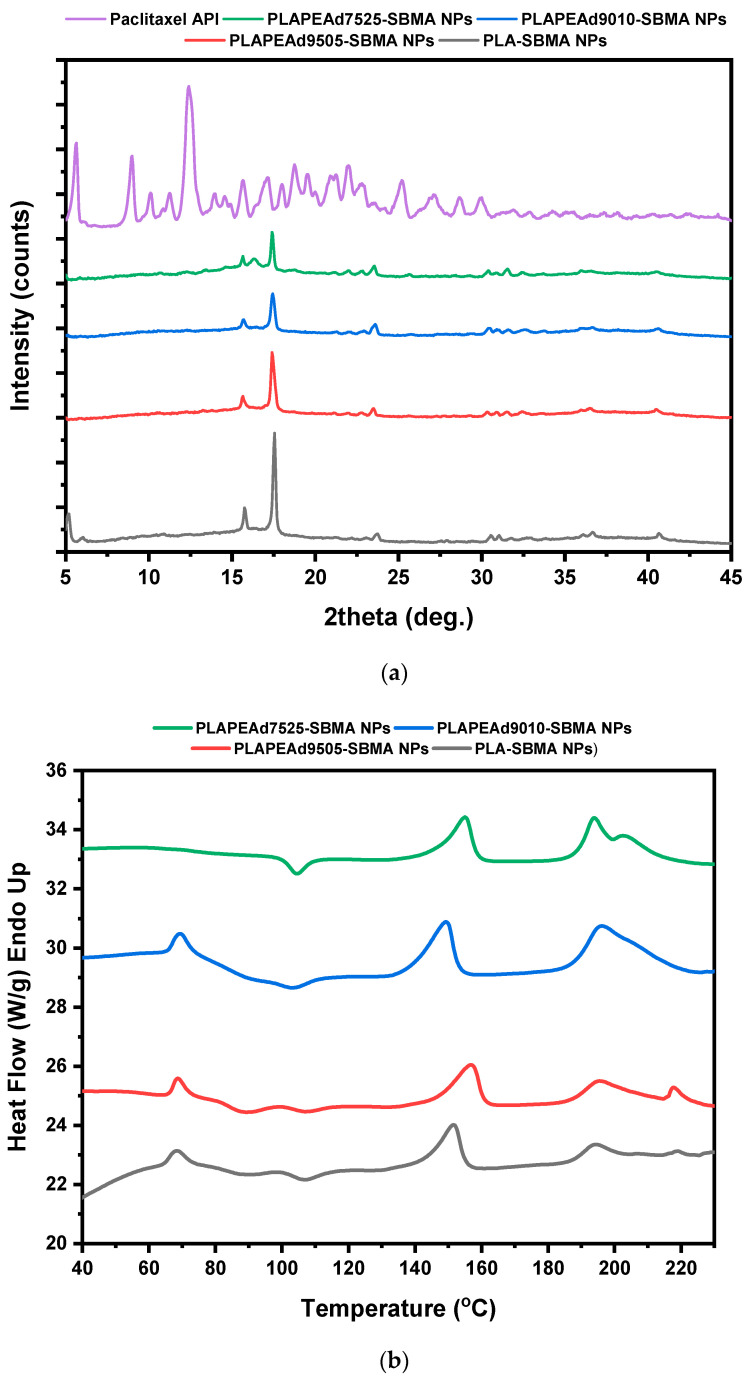
(**a**) XRD patterns and (**b**) DSC thermograms of drug-loaded nanoparticles.

The amorphous dispersion of the drug within the polymeric matrix is a highly desirable outcome, as it is associated with enhanced aqueous solubility, faster dissolution, and improved bioavailability, particularly important for poorly soluble drugs like paclitaxel. Additionally, the absence of crystalline PTX domains contributes to uniform release behavior from the nanoparticle matrix. To investigate the physical state of PTX within the zwitterionic nanoparticles and evaluate any physical transformations during particle formation, XRD and DSC were performed (Figure 9). 

The XRD pattern of pure PTX exhibits sharp and high intensity diffraction peaks between 5° and 25° 2θ, characteristic of its highly crystalline nature (Figure 9a). In contrast, the XRD diffractograms of all drug-loaded nanoparticles lacked any distinct crystalline reflections attributable to PTX. Instead, they showed broad halos typical of amorphous materials, dominated by the semi-crystalline PLA matrix, whose characteristic diffraction bands were significantly weakened or broadened compared to the neat copolymers (Figure 2a). This clear absence of PTX-specific crystalline peaks confirms that paclitaxel was incorporated in a molecularly dispersed or amorphous state within the polymer matrix [52].

Upon closer analysis of the comparative DSC thermograms (Figure 9b), a weak endothermic peak near 200–205 °C was observed in select formulations (PLA-SBMA and PLAPEAd9505-SBMA), suggesting the presence of a small fraction of crystalline paclitaxel. However, the peak was significantly attenuated compared to that of pure PTX, which typically displays a sharp melting endotherm around 217 °C, confirming that the largest amount of the drug is encapsulated in the amorphous state. In addition, a broad endothermic event around ~70 °C was visible in all thermograms except PLAPEAd7525-SBMA. This signal likely corresponds to a glass transition or cooperative relaxation event within the polymer–drug matrix. Its absence in the high-PEAd sample may be due to increased chain flexibility and phase disorder, which obscure this thermal feature. 

#### 3.2.4. pH-Responsive Drug Release Profiles

**Figure 10:**
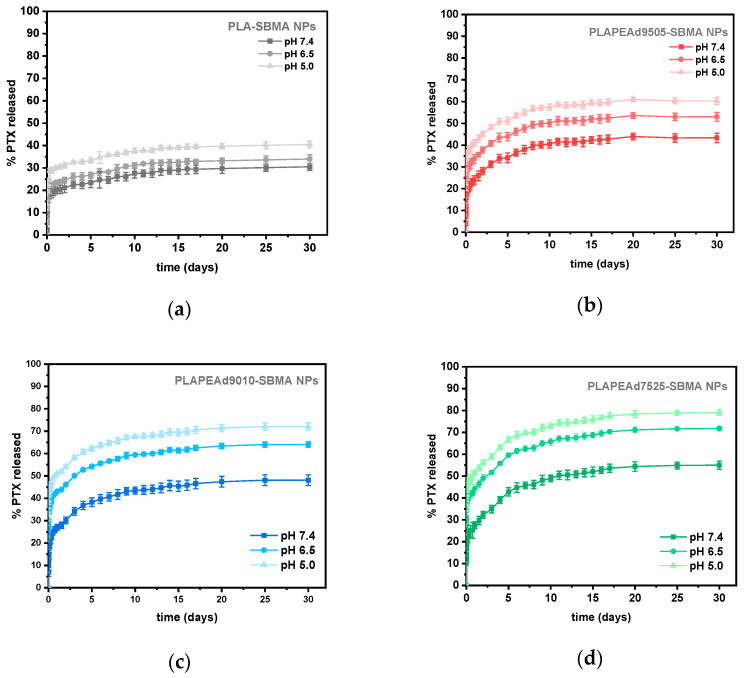
(**a**–**d**) In vitro release profiles of paclitaxel from zwitterionic nanoparticles at pH 7.4, 6.5, and 5.0 over 30 days, demonstrating a pH-responsive behavior across all formulations.

The in vitro release of paclitaxel (PTX) from the zwitterionic nanoparticles was investigated under physiologically relevant (pH 7.4) and tumor-mimicking acidic conditions (pH 6.5 and 5.0) over a 30-day period (Figure 10). In all cases, a biphasic release pattern was revealed, characterized by an initial burst release phase during the first 48 h, followed by a prolonged, sustained release phase. This dual-phase behavior is typical of polymeric nanocarriers, where surface-associated drug diffuses rapidly, while the remaining encapsulated drug is gradually released via matrix erosion and diffusion through the polymer network [53]. 

Table 3 summarizes the drug loading and encapsulation efficiency (EE) of the various nanoparticle formulations. PLA-SBMA nanoparticles achieved the highest EE (~58.3%), likely due to their tighter molecular packing and higher crystallinity, which restrict drug diffusion during nanoprecipitation and favor entrapment. In contrast, PLAPEAd7525-SBMA exhibited the lowest EE (~33.9%), correlating with its soft, amorphous character and broader molecular weight distribution, which may facilitate drug leakage during emulsification and solvent removal.

It should be noted that while the general trend shows decreasing encapsulation efficiency with increasing PEAd content, the value for PLAPEAd9010-SBMA (49.7%) was slightly higher than that of PLAPEAd9505-SBMA (42.6%). This deviation is within the variability of our measurements, and no formal statistical comparison was made. Such non-monotonic differences likely reflect the interplay of particle size, matrix composition, and interfacial drug retention during emulsification, and would require additional replicate studies with statistical analysis to confirm their significance.

In general, all formulations displayed strong pH-dependent release behavior, with accelerated PTX diffusion under acidic conditions, validating the stimuli-responsive design of the system. At pH 7.4, cumulative release values ranged from ~30% for PLA-SBMA to ~55% for PLAPEAd7525-SBMA, indicating moderate but formulation-dependent matrix permeability. PLA-SBMA nanoparticles released the lowest amount of drug across all conditions, reflecting their semicrystalline structure, hydrophobic surface, and high hydrolytic stability discussed earlier. In contrast, PLAPEAd7525-SBMA released ~54% of its PTX payload at pH 7.4 and up to ~79% at pH 5.0, demonstrating a more porous, rapidly degrading matrix (65–83% mass loss at pH 7.4 and 5.0, respectively) consistent with its high PEAd content (25%) and low crystallinity. PLAPEAd9010-SBMA followed a similar trend, with ~48% release at pH 7.4 and ~72% at pH 5.0. Its large size and relatively low molecular weight facilitated matrix destabilization and drug release under acidic conditions, whilst PLAPEAd9505-SBMA exhibited intermediate behavior, releasing ~43% of PTX at pH 7.4 and ~52% at pH 5.0. Its higher molecular weight and lower degradation rate (mass loss ~34% at pH 5.0) slowed drug release, offering a more controlled profile. 

Across all samples, the most significant increase in release occurred at pH 5.0, where acidic hydrolysis of ester bonds in PLA and PEAd segments most likely triggered matrix erosion, enhanced chain mobility, and facilitated drug diffusion. It is essential also to note that polymer molecular weight can also influence nanoparticle formation and drug retention during emulsification; lower-Mn copolymers may harden faster and reduce interfacial drug loss but tend to degrade more rapidly under acidic conditions. This dual effect is consistent with the faster release profiles observed for PEAd-rich, low-Mn formulations [54]. Moreover, in the context of in vivo performance, the faster-degrading, lower-Mn backbone could shorten long-term structural persistence, yet zwitterionic SBMA confers antifouling/stealth hydration layers that mitigate opsonization and promote circulation stability; factors favorable to exposure-selective release at acidic tumor sites [55].

The combination of SBMA-mediated hydrophilicity, PEAd-induced softness, and acid-triggered degradation generated a synergistic effect that enabled site-specific release behavior, minimizing drug leakage in systemic conditions while promoting efficient delivery at the simulated tumor site.

### 3.3. Evaluation of Biocompatibility

#### 3.3.1. Hemocompatibility Assessment

Evaluating the biocompatibility of newly designed nanoparticle systems is critical for their safe translation into biomedical applications. Hemocompatibility serves as a primary indicator of how such materials interact with the circulatory system upon intravenous administration, since red blood cells (RBCs) are the first cellular components that encounter the injected nanoparticles, thus any damage to their membranes associated with material toxicity can result in hemolysis [56]. In this sense, hemolysis assays are an essential first step to evaluating preclinical safety.

**Figure 11:**
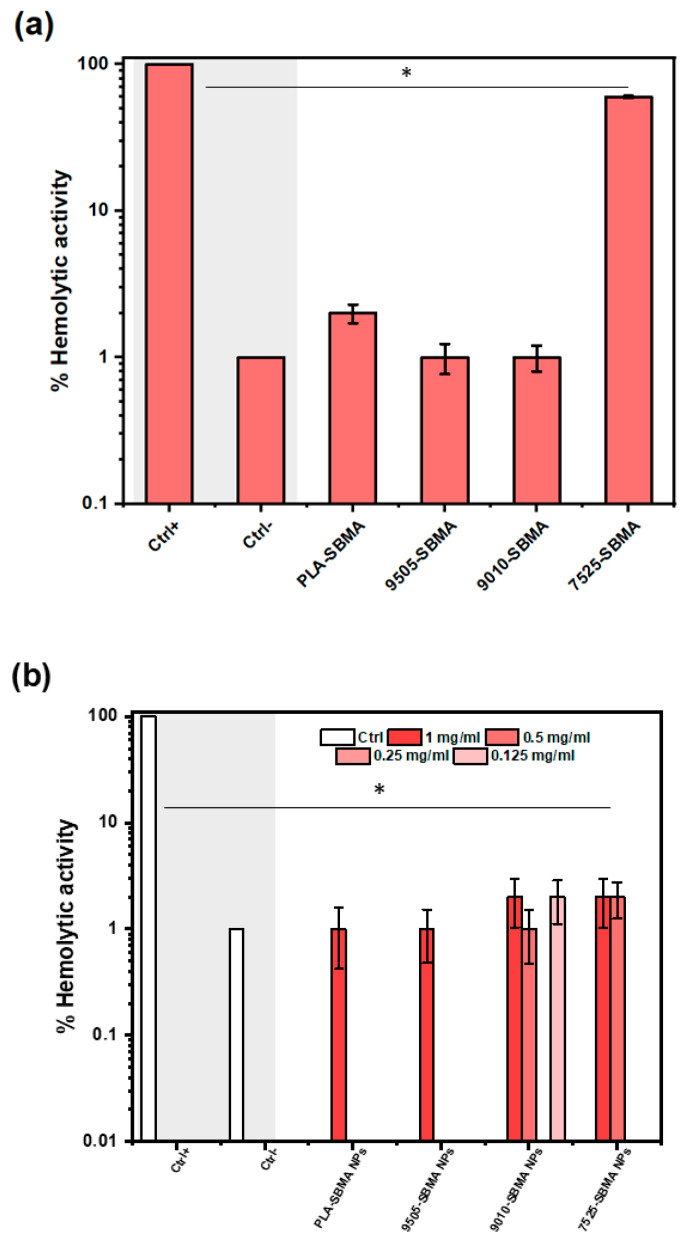
Hemolysis percentages for (**a**) the four neat zwitterionic copolymers, and (**b**) different nanoparticle concentrations (0.125, 0.25, 0.5 and 1 mg/mL) of each formulation at body temperature (37 °C), after 24 h incubation with RBCs. The symbol * represents the statistically significant differences between each compound and the positive control group (Ctrl+).

As shown in Figure 11a, the neat zwitterionic copolymers exhibited very low or negligible hemolytic activity after 24 h incubation with RBCs, with hemolysis percentages below 2% for PLA-SBMA, PLAPEAd9505-SBMA, and PLAPEAd9010-SBMA. These results indicate excellent compatibility with red blood cell membranes, largely attributed to the zwitterionic SBMA moieties, known for their non-fouling and non-thrombogenic characteristics. Interestingly, PLAPEAd7525-SBMA exhibited significant hemolytic activity (~60%), far exceeding the acceptable limit of 5%, as defined by ISO 10993-4 standards. This behavior likely stems from its high PEAd content, which may increase surface hydrophobicity and disrupt RBC membranes via direct amphiphilic interactions. Additionally, the copolymer’s low crystallinity, broad molecular weight distribution, and potentially incomplete surface shielding by SBMA moieties may contribute to its cytotoxic behavior. 

Interestingly, this effect was effectively suppressed upon nanoparticle formulation, suggesting that nanoencapsulation masks these hemolytic domains and stabilizes the polymer interface, thereby restoring hemocompatibility (Figure 11b). Generally, all drug-loaded nanoparticles tested at various concentrations (0.125–1 mg/mL) exhibited dose-independent hemolysis values well below the 5% threshold, confirming their blood compatibility and suitability for intravenous administration suggesting the use of low dose concentrations (0.125–0.5 mg/mL). These results further underline the stealth properties of zwitterionic SBMA segments, which are known to resist protein adsorption and immune cell recognition, thereby enhancing blood compatibility and circulation stability [51].

#### 3.3.2. In Vitro Cytotoxicity

An ideal delivery system should selectively act upon cells of interest, while minimizing toxicity to normal cells, therefore assessing the cytotoxicity of the nanocarriers is essential to ensure their therapeutic efficacy against cancer cells and biocompatibility with healthy tissues [57]. In this context, cytotoxicity assays can provide essential information regarding safety and selectivity. To this end, two representative cell lines were selected: the MDA-MB-231 human breast cancer line, a well-characterized, triple-negative, highly invasive tumor model; and immortalized mouse embryonic fibroblasts (iMEFs), which serve as a standard model of non-cancerous, healthy cells.

**Figure 12:**
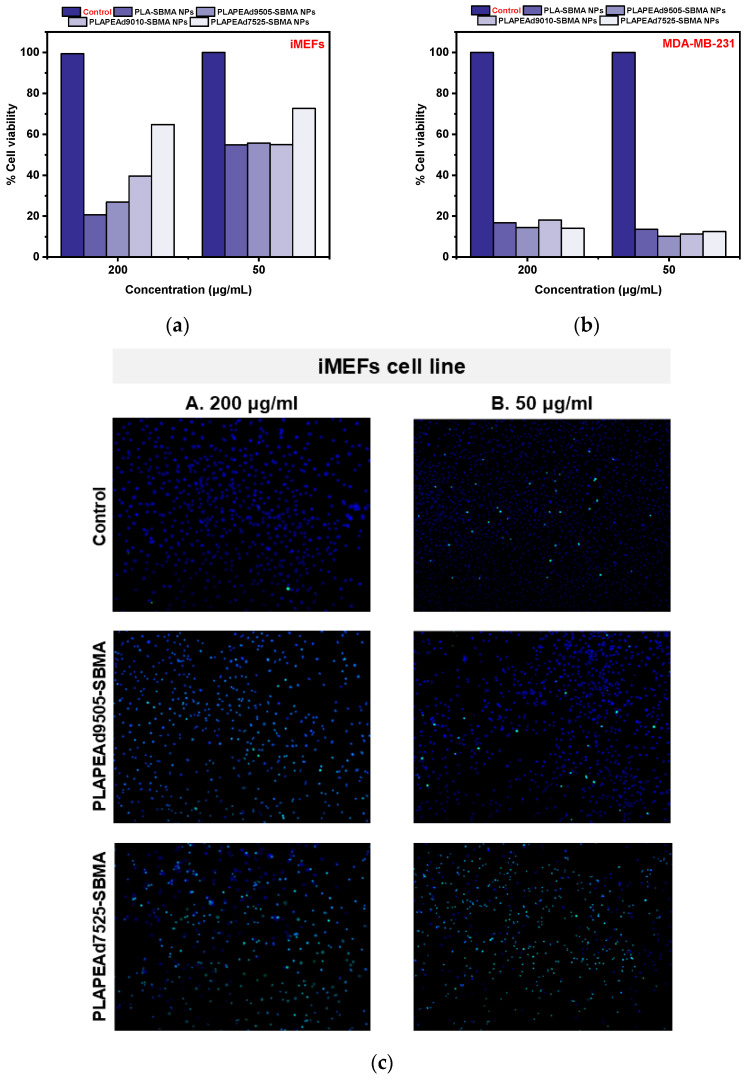
% Cell viability assay of (**a**) iMEF and (**b**) MDA-MB-231 cells treated with nanoparticle extracts, and (**c**) Fluorescent microscopy images upon iMEF cell line treatment with PLAPEAd9505-SBMA and PLAPEAd7525-SBMA zwitterionic nanoparticles at two different concentrations (A: 200 µg/mL, B: 50 µg/mL), indicating nuclear staining with Hoechst and CellTox™ Green fluorescence.

As presented in Figure 12a,b, treatment with nanoparticle extracts at a 1:5 dilution (200 μg/mL) for 18 h led to a strong cytotoxic response in MDA-MB-231 cells, with > 80% cell death across all formulations, confirming effective intracellular drug delivery and the therapeutic potential of the encapsulated paclitaxel. In contrast, the same treatment caused varied cytotoxicity in iMEFs, particularly low for samples with little or no amount of PEAd in their structure (i.e., 20.66% for PLA-SBMA and 64.79% for PLAPEAd9505-SBMA), and improved for formulations richer in PEAd segments (i.e., 39.6% for PLAPEAd9010-SBMA and 64.79% for PLAPEAd7525-SBMA). At a 1:20 dilution (50 μg/mL), the selectivity improved further, with MDA-MB-231 viability still significantly suppressed, while iMEF viability exceeded 50% for all formulations, supporting a dose-dependent and cell-type-specific cytotoxic profile. The improved viability of iMEFs in the presence of PEAd-rich nanoparticles may reflect not only reduced non-specific interactions but also enhanced pH-responsive behavior and matrix softness, which together contribute to better biocompatibility without compromising therapeutic efficacy. The results here correlate also very well with the release kinetics and degradation data discussed previously; the PLAPEAd9010-SBMA and PLAPEAd7525-SBMA nanoparticles, which showed faster PTX release at acidic pH and higher degradation, demonstrated the most pronounced cancer cell killing, whereas PLA-SBMA, which demonstrated a slower release, still maintained effective cytotoxicity, possibly due to the initial burst release phase.

Beyond this explanation, the improved selectivity observed for PEAd-rich nanoparticles (PLAPEAd9010-SBMA, PLAPEAd7525-SBMA) is likely not solely attributable to their faster, pH-triggered degradation and release at acidic tumor conditions. The incorporation of flexible PEAd segments may also increase surface hydrophilicity and reduce nonspecific interactions with healthy fibroblasts, thereby enhancing compatibility beyond the antifouling effect of SBMA. Together, these factors suggest that both degradation-driven kinetics and inherent compositional properties contribute to the lower iMEF toxicity observed. Further studies (e.g., protein adsorption or cellular uptake assays) would be required to confirm these mechanisms.

Finally, microscopic analysis of iMEFs treated with PLAPEAd9505-SBMA and PLAPEAd7525-SBMA nanoparticles at concentrations of 200 μg/mL and 50 μg/mL revealed distinct dose-dependent effects on cell viability (Figure 12c). Interestingly, CellTox™ Green fluorescence, which marks loss of membrane integrity (therefore late-apoptosis), was more pronounced at the lower concentration (50 μg/mL). This observation likely reflects the presence of more adherent, membrane-compromised cells at the lower dose, whereas at 200 μg/mL, many damaged cells may have undergone rapid detachment or complete lysis, thus escaping detection. Supporting this, Hoechst nuclear staining was visibly reduced at 200 μg/mL, suggesting significant loss of intact nuclei due to advanced apoptosis or necrosis. In contrast, cells treated at 50 μg/mL retained clearer nuclear morphology with detectable chromatin, despite showing membrane damage.

## 4. Conclusions

In this study, a series of zwitterionic copolymers based on PLA/PEAd matrices grafted with SBMA moieties was successfully developed and characterized for pH-responsive, tumor-targeted drug delivery applications. The resulting copolymers demonstrated tunable physicochemical properties depending on PEAd content, including reduced crystallinity, enhanced hydrophilicity, and acid-triggered degradation behavior. Paclitaxel-loaded zwitterionic nanoparticles exhibited controlled particle size, high drug encapsulation efficiency, and strongly pH-dependent release kinetics, particularly effective under acidic conditions mimicking the tumor microenvironment. Among all tested formulations, PLAPEAd7525-SBMA showed the most rapid drug release and degradation at pH 5.0, whereas PLA-SBMA nanoparticles exhibited slower release and greater structural stability. Moreover, all nanoparticles exhibited excellent hemocompatibility and demonstrated selective cytotoxicity against MDA-MB-231 cancer cells with reduced effects on healthy fibroblasts, especially at lower nanoparticle concentrations. This improved selectivity of PEAd-rich nanoparticles appears to arise from a combination of faster, pH-triggered degradation kinetics and inherent biocompatibility differences introduced by the flexible PEAd segments. Our findings confirm the potential of zwitterionic PLA/PEAd-SBMA nanoparticles as biodegradable platforms for controlled and selective chemotherapy.

## Figures and Tables

**Table 1:** GPC results for the zwitterionic PLA/PEAd-SBMA copolymers, indicating number-average molecular weight (Mn), weight-average molecular weight (Mw), and polydispersity index (PDI) values.

Sample	Mn (g/mol)	Mw (g/mol)	PDI
PLA-SBMA	4400	5600	1.28
PLAPEAd9505-SBMA	4900	6700	1.38
PLAPEAd9010-SBMA	3200	3800	1.17
PLAPEAd7525-SBMA	4500	6600	1.48

**Table 2:** Average particle size (nm) of paclitaxel-loaded zwitterionic nanoparticles measured by DLS at different pH conditions (7.4, 6.5, and 5.0).

NPs Sample	pH Value		
	7.4	6.5	5.0
PLA-SBMA	220 ± 24	242 ± 32	215 ± 18
PLAPEAd9505-SBMA	268 ± 33	283 ± 41	245 ± 26
PLAPEAd9010-SBMA	565 ± 48	608 ± 39	271 ± 34
PLAPEAd7525-SBMA	476 ± 29	524 ± 49	247 ± 21

**Table 3:** % Drug loading and EE values for all studied formulations. Note: The apparent deviation between PLAPEAd9010-SBMA and PLAPEAd9505-SBMA values falls within experimental variability; no formal statistical comparison was performed.

NPs Sample	% Drug Loading	% EE
PLA-SBMA	14.2 ± 0.86	58.3 ± 2.41
PLAPEAd9505-SBMA	12.78 ± 1.24	42.6 ± 1.85
PLAPEAd9010-SBMA	13.41 ± 0.92	49.7 ± 1.43
PLAPEAd7525-SBMA	9.83 ± 1.58	33.9 ± 1.67

## Data Availability

The original contributions presented in this study are included in the article and Supplementary Material. Further inquiries can be directed to the corresponding authors.

## Supplementary Materials

The following supporting information can be downloaded at: https://www.mdpi.com/article/10.3390/polym17182495/s1, Figure S1: ATR spectra of the neat PLA/PEAd copolymers; Table S1: Molecular weight values for the neat copolymers determined by GPC; Figure S2: TGA and DTG curves of the synthesized neat copolymers.
