# Supplementary material for: Biodegradable Zwitterionic PLA-Based Nanoparticles: Design and Evaluation for pH-Responsive Tumor-Targeted Drug Delivery
Source: Polymers (Basel). 2025 Sep 16;17(18):2495. doi: 10.3390/polym17182495 (PMC12473292; doi:10.3390/polym17182495)
Supplement: Supplementary file 1 [file polymers-17-02495-s001.zip › polymers-3812391-supplementary.pdf]

polymers

Biodegradable Zwitterionic PLA-based Nanoparticles: Design and Evaluation for pH-Responsive Tumor-Targeted Drug Delivery

SUPPLEMENTARY INFORMATION

1. Attenuated total reflection Fourier transform infrared (ATR-FTIR) spectroscopy

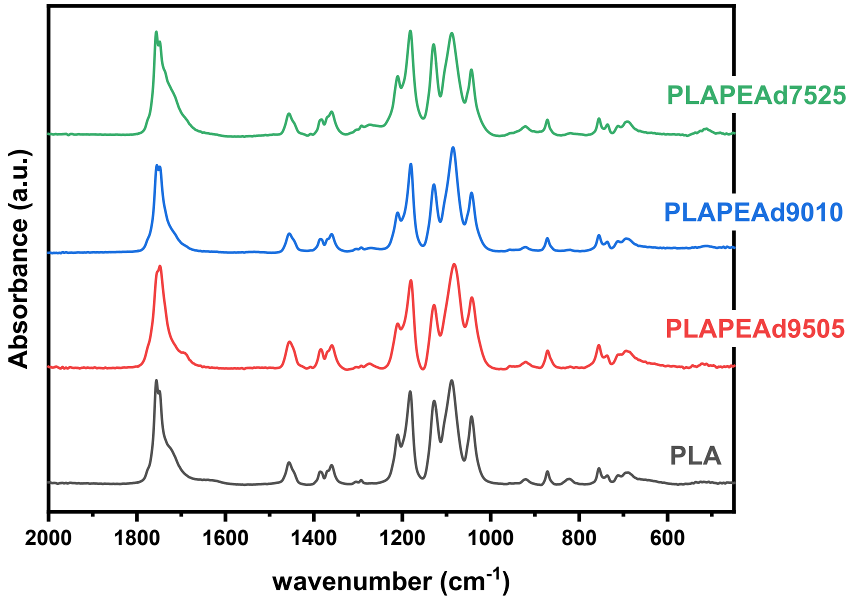

Figure S1. ATR spectra of the neat PLA/PEAd copolymers.

2. Gel Permeation Chromatography (GPC)

Table S1. Molecular weight values for the neat copolymers determined by GPC.

	Sample	Mn (g/mol)	Mw (g/mol)	PDI
Neat copolymers	PLA	42264	54090	1.3
	PLAPEAd9505	39000	53500	1.4
	PLAPEAd9010	34600	50800	1.5
	PLAPEAd7525	22700	31400	1.4
	PEAd	9500	18400	1.9

3. Thermogravimetric Analysis (TGA)

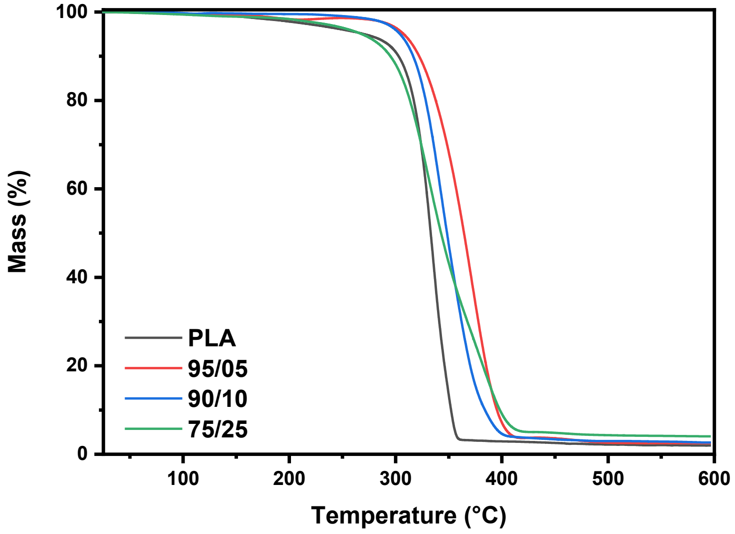

(a)

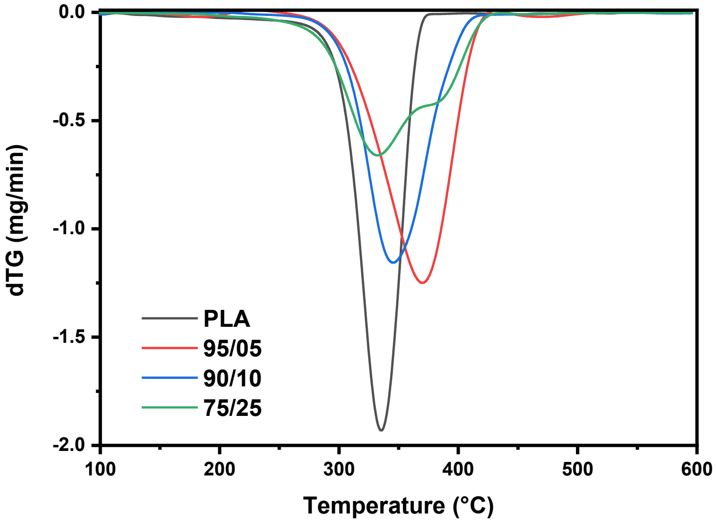

(b)

Figure S2. TGA and DTG curves of the synthesized neat copolymers
